# Engineered nanozyme immunomodulator for xerostomia treatment via regulating submandibular salivary gland

**DOI:** 10.1016/j.mtbio.2025.102555

**Published:** 2025-11-13

**Authors:** Xinyu Tao, Rui Zhao, Ye Fang, Minjie Chen, Cong Xu, Yujuan Zhu, Zhifeng Gu

**Affiliations:** a Department of Rheumatology, Research Center of Clinical Medicine, Research Center of Immunology, Affiliated Hospital of Nantong University, Medical School of Nantong University, Nantong, 226001, China; b Department of Biomedical Engineering, Columbia University, New York, NY, 10027, USA; c Department of Neurosurgery, Health Science Center, The First Affiliated Hospital of Shenzhen University, Shenzhen Second People’s Hospital, Shenzhen, 518035, China

**Keywords:** Nanoparticles, Cerium oxide nanozyme, Xerostomia, Targeting submandibular salivary gland, Immunotherapy

## Abstract

Xerostomia, or dry mouth, manifests as a symptom of hyposalivation. Current clinical management primarily relies on palliative therapies to alleviate symptoms. However, progressive salivary gland damage often leads to irreversible functional loss, severely compromising patients' quality of life. Consequently, there is an urgent need for therapeutics that can promote salivary gland repair and functional recovery. Herein, we developed a novel cerium-based nanozyme delivery system for hyposalivation immunotherapy. Following intravenous injection, the nanosystem preferentially accumulates in mouse submandibular glands, leveraging the reported affinity of its copolymer coating for muscarinic acetylcholine receptors. At the target site, the nanozyme scavenges reactive oxygen species by mimicking superoxide dismutase and catalase activities. This antioxidant action mitigates local inflammation and tissue edema, restores functional gland morphology, and ultimately enhances saliva secretion. Transcriptomic analysis further revealed that the treatment modulates multiple immune-related and inflammatory signaling pathways. Collectively, this cerium nanozyme-based immunomodulatory strategy represents a promising approach for treating hyposalivation and addressing progressive salivary gland injury.

## Graphical abstract

**Figure:**
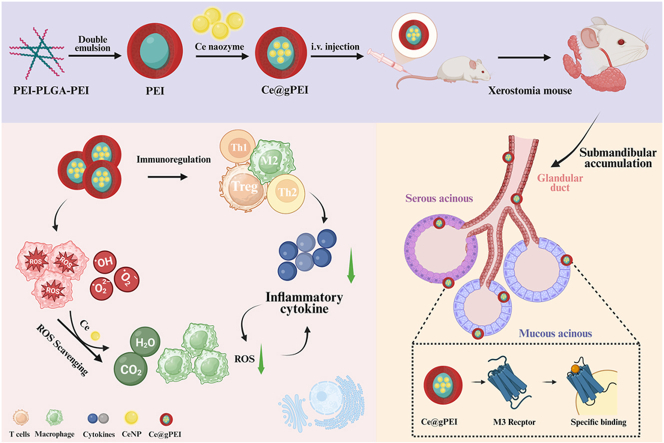

## 1 Introduction

Xerostomia, characterized by the subjective sensation of dry mouth, arises from insufficient saliva production, significantly impacting oral health and quality of life. Hyposalivation, the objective reduction in salivary flow, is the underlying cause of xerostomia [1,2]. The condition is multifactorial, involving both systemic and local factors. Systemic contributors include autoimmune disorders such as Sjögren's Disease (SjD), diabetes, Parkinson's disease, and aging, while local factors encompass medication side effects, head and neck radiotherapy, and certain lifestyle behaviors [[3], [4], [5], [6]]. Globally, the prevalence of xerostomia among adults and the elderly is approximately 22 % [6], with nearly all SjD patients experiencing this debilitating symptom [3]. Current clinical management of xerostomia remains predominantly palliative, relying on interventions such as saliva substitutes, non-steroidal anti-inflammatory drugs, pilocarpine, and traditional Chinese medicine [7,8]. Although these approaches offer symptomatic relief, they fail to reverse glandular dysfunction or halt disease progression. Moreover, the frequent administration of topical treatments like saliva substitutes can disrupt daily activities and reduce compliance, as patients must carry and apply them multiple times throughout the day. For systemic agents like pilocarpine and cevimeline, their efficacy is often limited to a subset of patients, and they can induce undesirable systemic side effects such as sweating, nausea, and urinary frequency. [[9], [10], [11]]. Therefore, there is a pressing need for therapies that not only alleviate symptoms but also address the underlying pathophysiology to restore salivary gland function. A growing body of evidence implicates oxidative stress as a prominent contributor to the development of xerostomia, characterized by an overproduction of reactive oxygen species (ROS) that aggravates salivary gland injury [[12], [13], [14]]. Infiltration of salivary tissues by macrophages and neutrophils exacerbates local oxidative stress by driving ROS production via NADPH oxidase, thereby amplifying inflammatory pathways and impairing secretory function [15]. These insights underscore the therapeutic potential of strategies that concurrently mitigate oxidative stress and modulate immune responses.

**Fig. 1:**
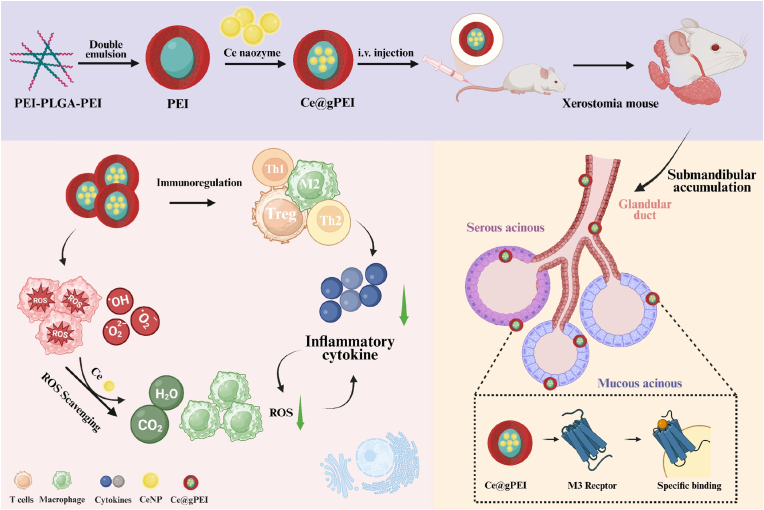
Schematic illustration of the engineered Ce@gPEI immunomodulator and its applications in the immunotherapy of xerostomia.

In this study, we proposed a nanozyme-based delivery system for the immunotherapy of xerostomia that preferentially accumulates in the salivary gland. (Fig. 1). Nanozymes represent a class of functional nanomaterials that mimic the catalytic behavior of natural enzymes, enabling them to facilitate biochemical reactions under physiological conditions. In contrast to conventional enzymes, their catalytic prowess originates from their nanoscale structural features rather than specific protein active sites [[14], [15], [16], [17]]. Moreover, these synthetic enzymes demonstrate superior stability, minimal immunogenicity, and cost-effective synthesis, positioning them as attractive candidates for biomedical applications [18].More importantly, the catalytic properties of nanozymes can be precisely tuned through the integration of delivery carriers and surface modifications, thereby meeting diverse clinical and research requirements [19,20].In addition, designed for site-specific pharmacotherapy, targeted delivery platforms enable the directed transport of therapeutic agents to diseased tissues. Through precise control over release kinetics and duration, these systems facilitate sustained or stimuli-responsive drug liberation. Such an approach not only augments treatment efficacy but also enhances in vivo stability and reduces off-target side effects, ultimately leading to a more optimized and effective treatment outcome [[21], [22], [23]].Moreover, targeted drug delivery systems have manifested remarkable potential in the management of a diverse spectrum of diseases, spanning cardiovascular, neurological, inflammatory, and metabolic disorders [[24], [25], [26]]. By synergistically integrating the distinctive attributes of nanozymes with the capabilities of targeted drug delivery, it is highly anticipated that this convergence will yield substantial alleviation of xerostomia patient symptoms, heralding a new era of advanced therapeutic interventions.

Herein, we developed a novel nanozyme-based delivery system by integrating cerium oxide nanozyme (CeNP) and polyethylenimine-poly (lactic-co-glycolic acid) copolymer (PEI-PLGA), which could be applied to alleviate the symptoms of dry mouth via regulating the salivary glands. CeNP are encapsulated by polyethyleneimine (PEI), enabling the construction of a double-layer membrane spherical structure. The synthesized Ce@gPEI nanocomposites exhibit excellent biocompatibility. The hydrophilic segment of this structure is exposed on the surface, allowing it to specifically bind to acetylcholine receptors, thereby selectively accumulating in the submandibular salivary glands. Moreover, Ce@gPEI nanosystem exhibits remarkable reactive oxygen species (ROS) scavenging and anti-inflammatory impacts in macrophages. Using a xerostomia mouse model, Ce@gPEI can precisely accumulate into the submandibular gland, where they effectively mediate significant anti-inflammatory and antioxidant actions. The nanosystem not only alleviates tissue edema, but also restores the functional morphology of submandibular gland tissue, substantially augmenting saliva secretion, and effectively curbing the onset and progression of oral dryness. Transcriptome analysis has unveiled that the present nanosystem suppresses the inflammatory response by modulating multiple immune-related signaling pathways, including NOD-like receptor signaling pathway, IL-17 signaling pathway, and Th1/Th2 cells differentiation pathway. Notably, within the TNF signaling pathway, the expression of TNF is significantly down-regulated, which is essential for the inception and development of the inflammatory cascade. The study also reveals the up-regulation of bPRP, which is crucial for the secretion of both saliva and tears. Collectively, the engineered Ce@gPEI immunomodulator curbs the inflammatory response through the regulation of immune signaling pathways, ultimately leading to enhanced saliva secretion. These findings underscore the considerable potential of the Ce@gPEI nanosystem, with its targeted submandibular gland capability, as a promising therapeutic approach for xerostomia and salivary gland injury.

## 2 Results and discussion

**Fig. 2:**
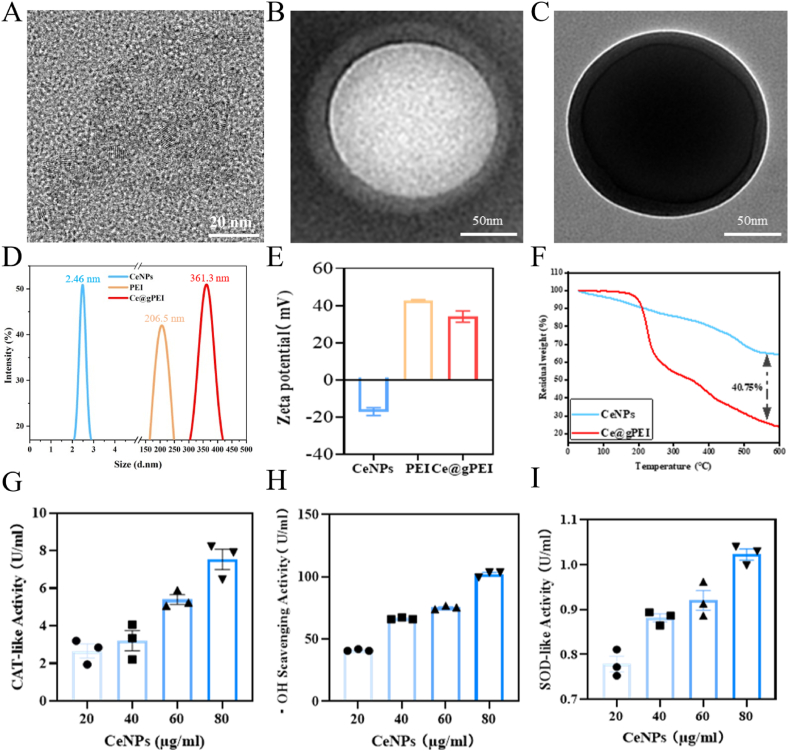
**Fabrication and Assessment of Ce@gPEI.** (A) HR-TEM images of cerium oxide nanozymes. Scale bar, 20 nm. (B–C) TEM images of PEI and CNP@ PEI nanozymes. Scale bar, 50 nm. (D) Size distributions of CeNPs, PEI and Ce@gPEI nanozymes. (E) Zeta potential of CeNPs, PEI and Ce@gPEI nanoparticles. (F) TGA curves of CeNPs and Ce@gPEI. (G–I) Etection of catalase (CAT), hydroxyl radical scavenging, and superoxide dismutase (SOD) mimicking activities of CeNPs.

In this study, CeNPs were synthesized through a two-step process. First, a complexation reaction occurred between cerium nitrate hexahydrate and disodium citrate, followed by a precipitation reaction triggered by the addition of ammonia water. Representative high-resolution transmission electron microscopy (HR-TEM) image showed the crystalline characteristics of CeNPs, with lattice spacing ranging from 0.303 to 0.316 nm (Fig. 2A). The amphiphilic PEI-based triblock copolymer molecules spontaneously self-assemble into spherical nanoparticles within water/oil/water (W/O/W) emulsions. Notably, their hydrophilic PEI segments fully exposed on the surface, enveloping the aqueous phase. Dynamic light scattering revealed that PEI nanoparticles had a mean hydrodynamic size of ∼206.5 nm and a zeta potential of +42.65 mV. Using a double-emulsion technique, we successfully synthesized Ce@gPEI nanocomposites. Following CeNP encapsulation, the average diameter increased to approximately 361.3 nm, accompanied by a decrease in zeta potential to +34.08 mV (Fig. 2D and E). TEM was employed to characterize the morphology of Ce@gPEI nanoparticles (Fig. 2B–C). The images showed that the incorporation of CeNPs did not disrupt the structural integrity of the PEI nanoparticles. The lattice of CeNPs contains oxygen vacancies, which increase the surface area and the number of active sites, thereby enhancing the material's catalytic activity [27]. XPS analysis revealed that Ce@gPEI exhibited an identical Ce^3+^/Ce^4+^ (oxidation/reduction) ratio pattern to that of CeNPs (Figure S1), thereby confirming the co-existence of both oxidation states in Ce@gPEI. As depicted in Fig. 2F, the TGA profiles of CeNPs and Ce@gPEI were obtained under an air atmosphere in the temperature range of 30–600 °C. The TGA curve for Ce@gPEI exhibited continuous weight loss (from 100 % to 23 %) within the temperature interval of 30–600 °C.This weight loss was attributed to the decomposition of adsorbed water and the degradation of Ce@gPEI itself. The remaining 23 % is attributed to the inorganic cerium content of Ce@gPEI. The results showed that PEI was successfully combined with bare ceria nanoparticles, and the content of the former was as high as 40.75 wt%.

Subsequently, we evaluated the multi-enzyme mimetic properties of CeNPs, encompassing catalase-like (CAT) activity for decomposing hydrogen peroxide, hydroxyl radical elimination capacity, and superoxide dismutase (SOD)-like function responsible for neutralizing superoxide anions.The results showed that CeNPs exhibited substantial dose-dependent activities for catalase mimicry (Fig. 2G), hydroxyl radical scavenging (Fig. 2H), and superoxide dismutase mimicry (Fig. 2I). These findings collectively demonstrate the Ce@gPEI nanosystem's ability to efficiently scavenge diverse reactive oxygen species (ROS), highlighting its significant antioxidant capacity.

**Fig. 3:**
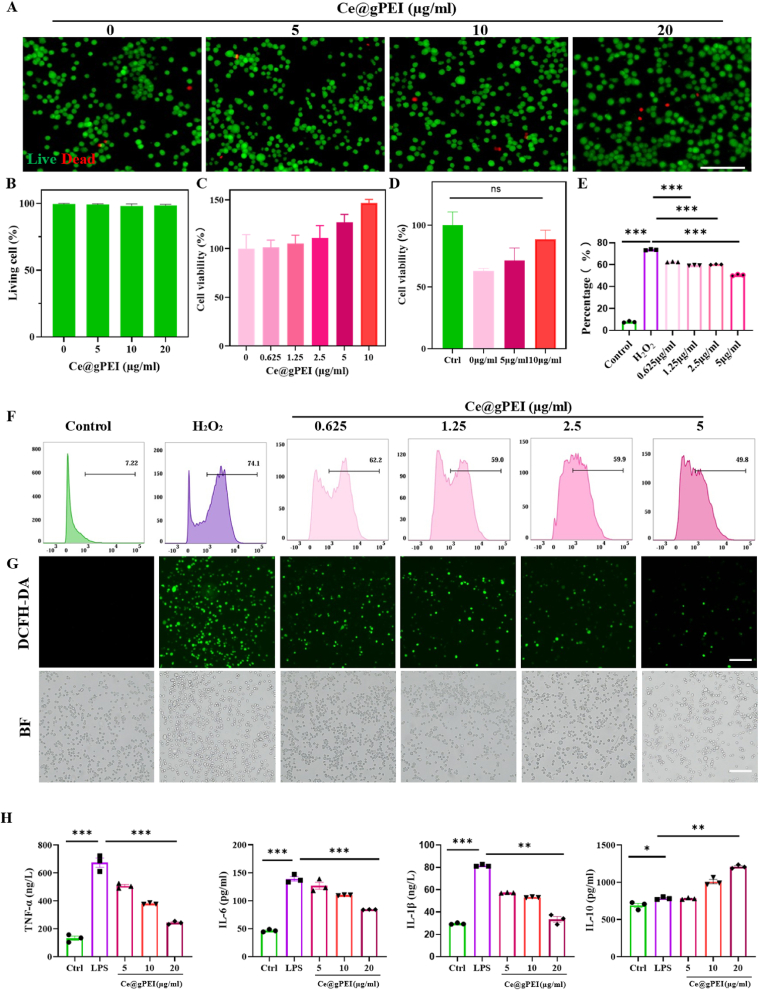
**Biocompatible and functional evaluation of Ce@gPEI nanocomposite.** (A) Fluorescence images of Calcein/PI staining. Scale bar, 50 μm. (B) Quantitative analysis of viable cells across different groups. (C) Viability of RAW264.7 macrophages following 24-h exposure to varying concentrations of Ce@gPEI for 24 h. (D) Quantitative assessment of RAW264.7 cell viability after H_2_O_2_ stimulation (600 μM) and treatment with increasing concentrations of Ce@gPEI. (E) Statistical summary of flow cytometry data shown in (F), representing intracellular ROS levels in H_2_O_2_-stimulated RAW264.7 macrophages after treatment with Ce@gPEI. (G) Representative DCF fluorescence (green) and corresponding brightfield (BF) images of RAW264.7 cells showing intracellular ROS levels after H_2_O_2_ exposure and Ce@gPEI treatment. Scale bar, 100 μm. (H) Concentrations of TNF-α, IL-6, IL-1β, and IL-10 in cell culture supernatants across experimental groups (n = 3). ∗*P* < 0.05, ∗∗*P* < 0.01, ∗∗∗*P* < 0.001.

The biocompatibility of Ce@gPEI is a prerequisite for its clinical translation. To evaluate this, RAW264.7 macrophages were treated with varying concentrations of Ce@gPEI. As shown in Fig. 2A–B, a large number of viable cells (stained green) were observed, with only a minimal number of dead cells detected, even at high concentrations of Ce@gPEI. Similar results were obtained when HUVECs were exposed to the same treatment regimen (Figure S2A–B). Subsequently, the cytotoxicity of Ce@gPEI at different concentrations was quantitatively assessed using the CCK-8 assay on both RAW264.7 macrophages and HUVECs. As shown in Fig. 3C and Figure S2C, within the tested concentration range, both cell lines demonstrated excellent tolerance to the nanocomposites. Notably, cell viability remained high even with increasing Ce@gPEI concentrations, providing further evidence of the favorable biocompatibility of Ce@gPEI.

Although the enzyme-like activity of the Ce@gPEI nanosystem has been confirmed, its efficacy within the cellular environment remains to be elucidated. To assess the cytoprotective effects of Ce@gPEI, Initially, we established that the optimal hydrogen peroxide concentration for stimulation was 600 μM (Figure S2D). Compared to the control group (Fig. 3D), hydrogen peroxide stimulation significantly reduced cell viability, whereas pretreatment with Ce@gPEI effectively reversed this trend. Consistent results were observed in HUVECs (Figure S2E). To further investigate the ROS scavenging capability of Ce@gPEI, flow cytometry has been performed to quantify intracellular ROS levels. As shown in Fig. 3E–G, within a certain concentration range, Ce@g PEI exhibited dose-dependent ROS inhibition, confirming its antioxidant efficacy in cellular models. Given the complex relationship between inflammation and ROS, wherein inflammation triggers ROS production, and excessive ROS exacerbates the inflammatory response, we next explored the immunomodulatory properties of Ce@gPEI. ELISA assay was carried out to measure key inflammation-related cytokines, including TNF-α, IL-6, IL-1β and IL-10 (Fig. 3H). The results showed that increasing concentrations of Ce@gPEI significantly reduced the levels of pro-inflammatory cytokines (TNF-α, IL-6 and IL-1β) while enhancing the production of the anti-inflammatory cytokine IL-10. These results suggest that Ce@gPEI plays a crucial role in regulating the inflammatory response by suppressing pro-inflammatory mediators and promoting the release of anti-inflammatory cytokines.

**Fig. 4:**
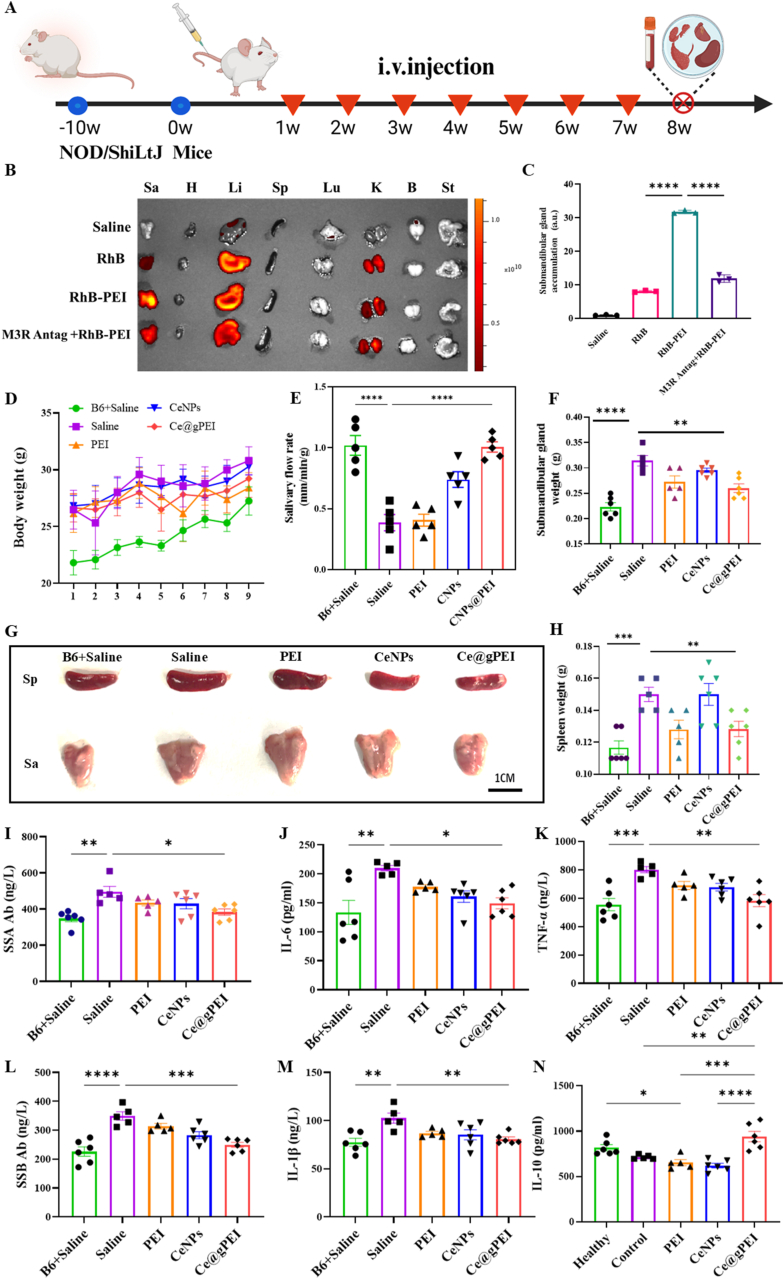
***In vivo* therapeutic efficacy of Ce@gPEI in a mouse model.** (A) Schematic illustration of the experimental design, depicting the timeline for establishing the NOD mouse xerostomia model, weekly intravenous treatments, and salivary flow rate measurements at week 18. (B) Ex vivo fluorescence imaging of key organs harvested 8 h post-injection with free RhB, RhB-PEI, or RhB-PEI following pre-treatment with the M3 receptor antagonist Tiotropium Bromide. Sa, submandibular salivary gland; H, heart; Li, liver; Sp, spleen; Lu, lung; K, kidney; B, brain; St, stomachs. (C) Quantitative analysis of fluorescence intensity in the submandibular gland (n = 3), confirming targeted nanoparticle accumulation. (D) Weekly body weight monitoring throughout the treatment period. (E) Saliva flow rates measured across treatment groups (n = 5). (F) Submandibular gland weights normalized to body mass (n = 5). (G) Representative images of submandibular glands and spleens from each treatment group. (H) Spleen weights normalized to body mass (n = 5). (I–N) Serum cytokine levels (TNF-α, IL-6, IL-1β, IL-10) quantified via ELISA (n = 5). ∗*P* < 0.05, ∗∗*P* < 0.01, ∗∗∗*P* < 0.001.

To explore the practical value of Ce@gPEI nanosystem, a xerostomia model was established using NOD mouse, which naturally develop oral dryness by approximately 13 weeks of age. Via a sequence of initial dose-ranging trials, the optimal therapeutic dosage of Ce@gPEI was determined to be 30 mg/kg (Figure S3-S4). The treatment regimen consisted of an 8-week intravenous administration via the tail vein, initiated at week 11. Salivary flow rates were measured at week 18, after which the mice were euthanized, and tissue samples were collected for in-depth analysis. (Fig. 4A). The trimethylamine groups exposed on the surface of Ce@gPEI nanoparticles are hypothesized to exhibit specific affinity for the muscarinic M3 acetylcholine receptor (M3R), which is highly expressed in submandibular salivary glands of both murine and human origin and plays a pivotal role in regulating salivary secretion [23]. To validate the targeting efficiency and receptor specificity of the nanocomposite, in vivo fluorescence imaging was performed following intravenous administration of free Rhodamine B (RhB), Rhodamine B-labeled Ce@gPEI (RhB-Ce@gPEI), or RhB-Ce@gPEI after pre-treatment with the M3 receptor antagonist Tiotropium Bromide. Compared with free RhB, RhB-Ce@gPEI nanoparticles exhibited significantly enhanced accumulation in the murine submandibular gland (Fig. 4B and C). This targeted accumulation could be effectively abolished by M3 receptor blockade, indicating that the targeting is mediated specifically by the M3 receptor. Quantitative fluorescence analysis revealed that the signal intensity in the RhB-Ce@gPEI group was more than three-fold higher than that in the free RhB control group, strongly confirming the submandibular salivary gland-targeting capability of the nanosystem and its dependence on the M3 receptor pathway.

Throughout the 8-week treatment period, mouse body weight was monitored prior to each weekly intravenous injection (Fig. 4D). All groups exhibited consistent, age-appropriate weight gain, with no statistically significant differences observed between Ce@gPEI-treated and control groups. Histological examination of major organs (liver, kidney, heart, spleen and lung) from Ce@gPEI-treated mice demonstrated no significant pathological abnormalities compared to the control group (Figure S5). These results further validate the exceptional biocompatibility of the Ce@gPEI nanosystem, indicating minimal systemic toxicity or adverse effects on growth. Clinically, submandibular gland damage is characterized by reduced salivary flow rates and compensatory glandular hypertrophy, making these metrics for assessing xerostomia severity. Salivary flow rates were measured immediately prior to euthanasia, and submandibular gland weights were recorded post-mortem (Fig. 4E–G). The saline control group exhibited significantly reduced salivary flow accompanied by pronounced glandular hypertrophy, consistent with xerostomia pathophysiology. In contrast, Ce@gPEI treatment reversed these pathological changes, restoring salivary flow rates and normalizing glandular size. Furthermore, the therapeutic efficacy of Ce@gPEI was corroborated by longitudinal monitoring of drinking behavior, which revealed a significant reduction in weekly w ater consumption in treated mice compared to the xerostomia control group (Figure S6), indicating alleviation of polydipsia symptoms. Spleen size, an indicator of systemic inflammation and immune response, was also evaluated. Xerostomic mice displayed significant splenomegaly relative to healthy controls, indicative of chronic systemic inflammation. Ce@gPEI treatment effectively normalized splenic enlargement (Fig. 4H), suggesting that the nanosystem mitigates both local salivary gland pathology and systemic autoimmune manifestations associated with xerostomia.

To further explore immunomodulatory effects, ELISA assay was carried out to detect serum levels of SSA (anti-Ro antibody) and SSB (anti-La antibody) autoantibodies, hallmark serological markers of xerostomia. Ce@gPEI treatment significantly reduced the levels of both autoantibodies (Fig. 4I and J), indicating that it mitigates pathogenic autoimmune responses. Given that chronic salivary gland inflammation drives xerostomia progression, pro- and anti-inflammatory factors were analyzed. Consistent with in vitro experiments, Ce@gPEI treatment decreased expression of Inflammatory mediators (TNF-α, IL-1β, and IL-6) and elevated levels of the anti-inflammatory cytokine IL-10. These findings indicate that Ce@gPEI retains potent immunomodulatory and anti-inflammatory activities in vivo, underscoring its therapeutic potential for xerostomia.

**Fig. 5:**
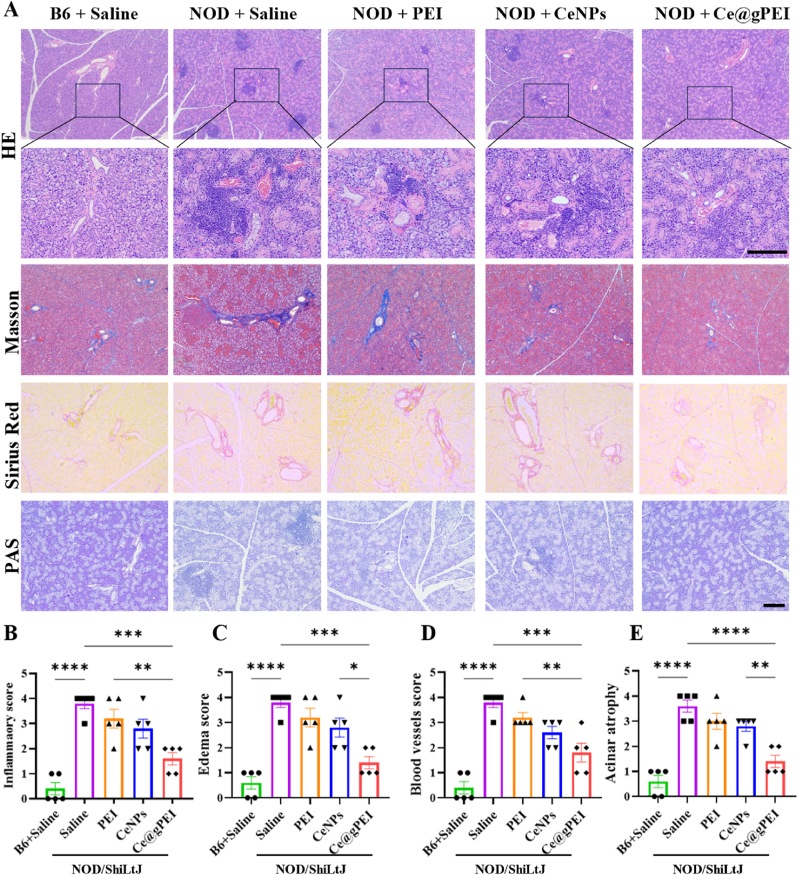
Histopathological characterization of submandibular glands in xerostomia mice after Ce@gPEI treatment. (A) Representative images of HE, Masson, Sirius Red, and PAS staining of submandibular gland tissue in different groups. Scale bar, 200 μm. (B–E) Inflammatory infiltration (B), tissue edema (C), vascular congestion (D), acinar atrophy. (E) Semi-quantitative analysis score in each experimental group. ∗*P* < 0.05, ∗∗*P* < 0.01, ∗∗∗*P* < 0.001.

To further explore the therapeutic efficacy of Ce@gPEI nanocomposites, we conducted histological evaluations on the submandibular glands (Fig. 5A). Statistical analysis of HE staining indicated that, compared with the control group, Ce@gPEI treatment significantly mitigated inflammatory cell infiltration, tissue edema, vascular congestion and acinar atrophy in submandibular gland tissue, with highly significant statistical differences (Fig. 5B–E). These results strongly show that Ce@gPEI plays a pivotal role in repairing and reconstructing both the morphology and function of the submandibular gland. Masson trichrome staining (collagen fibers stained blue) and Sirus Red staining (collagen fibers stained green) are commonly applied to discover collagen fibers in salivary gland tissues [28]. As presented in Fig. 5A, the submandibular gland tissue of xerostomia mice in the saline group exhibited a marked increase in collagen fibers, which densely encircled the ductal acini, in stark contrast to normal control mice. In contrast, after Ce@gPEI treatment, the amount of collagen fibers in the submandibular gland tissue decreased substantially, and the tissue structure reverted to a more physiologically normal state. PAS staining was also employed to visualize changes in mucin and glycogen accumulation within salivary gland acinus [29].Compared with normal mice, xerostomia mice showed a downward trend in PAS staining intensity in submandibular gland tissue, indicating reduced mucin and glycogen content. However, intravenous injection of Ce@gPEI effectively reversed this trend. Collectively, the histological analysis demonstrates that Ce@gPEI is highly effective in alleviating salivary gland damage.

**Fig. 6:**
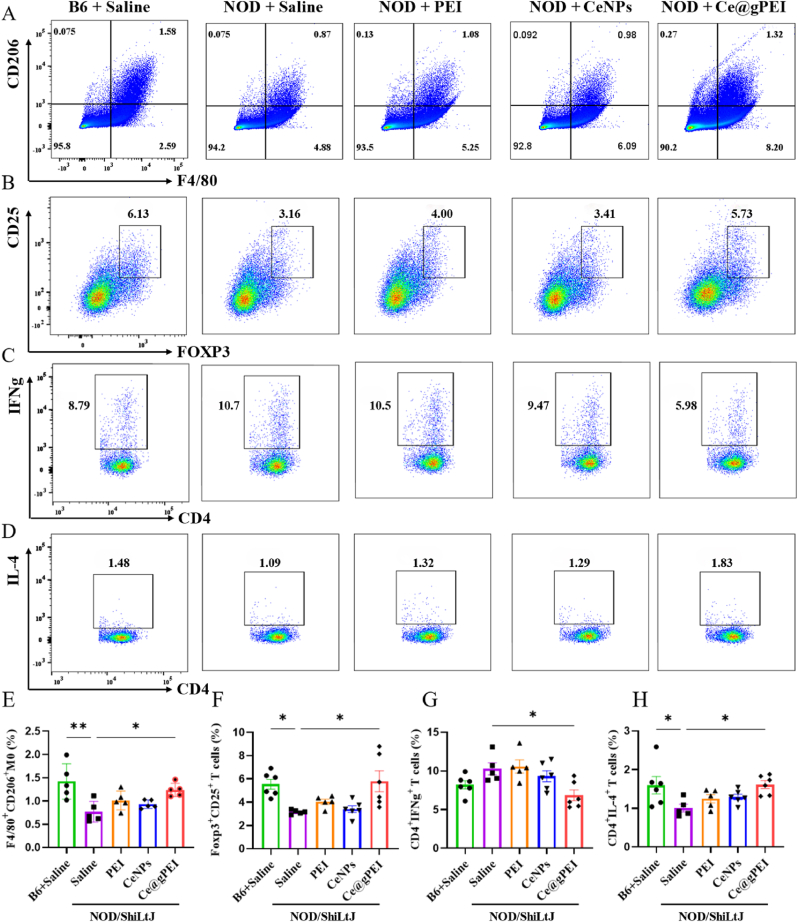
**Characterization of splenic immune cell populations in xerostomia mice with Ce@gPEI treatment.** (A–D) The proportion of M2 (A), Treg (B), Th1 (C) and Th2 (D) within the splenocyte populations in the B6+Saline, NOD + Saline, NOD + PEI, NOD + CeNPs and NOD + Ce@gPEI groups. (E–H) Quantitative analysis of the proportions of M2 (E), Treg (F), Th2 (H) across different groups by flow cytometry. N = 5, ∗*P* < 0.05, ∗∗*P* < 0.01, ∗∗∗*P* < 0.001.

Upon observing a statistically significant variation in spleen size across treatment groups, we hypothesized that Ce@gPEI might exert its therapeutic effects by modulating immune cell populations within the body. To test this hypothesis, flow cytometry was employed to analyze immune cell subsets in mouse spleens, including F4/80+ CD206+ M2 macrophages, Foxp3+ CD25^+^ Treg cells, CD4^+^ IFNγ+ Th1 cells, and CD4^+^ IL-4+ Th2 cells (Fig. 6A–D, and S7). Our results indicated that Ce@gPEI treatment significantly increased the proportions of anti-inflammatory cell subsets in the spleen, including M2 macrophages (0.87 % vs 1.32 %), Treg cells (3.16 % vs 5.73 %), and Th2 cells (1.09 % vs 1.83 %). M2 macrophages are recognized as key players in the pathogenesis of xerostomia, and reductions in their quantity or functional impairment are closely associated with the inflammatory responses and glandular damage characteristic of the disease. Treg cells, a subset of immunosuppressive T cells, alleviate salivary gland inflammation in xerostomia by inhibiting the activation of pro-inflammatory immune cells, such as Th1 and Th17 cells. Th2 cells, meanwhile, contribute to restoring immune homeostasis and mitigating inflammation through the secretion of cytokines, including IL-4 and IL-5. In contrast, Th1 cells secrete IFN-γ, a cytokine involved in driving cell-mediated immune responses and promoting inflammatory processes [14,30]. These findings suggest that Ce@gPEI exerts therapeutic effects in xerostomia by modulating the balance of immune cell populations, thereby restoring immune homeostasis and alleviating disease sym ptoms.

**Fig. 7:**
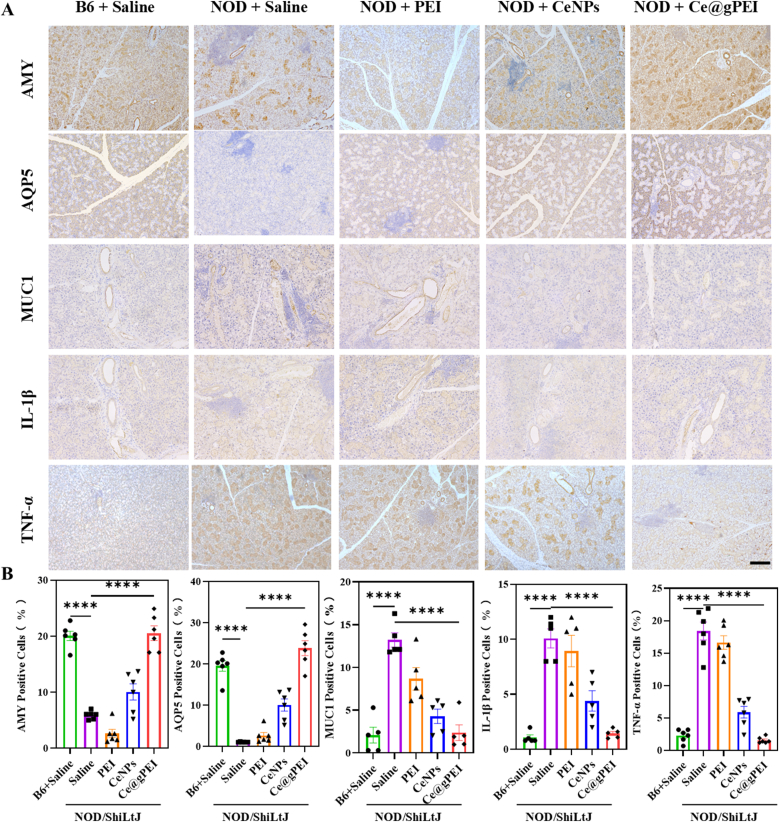
**The pathological change of submandibular gland.** (A) AMY, AQP5, MUC1, IL-1β, and TNF-αimmunohistochemistry in each group. Scare bar, 100 μm. (B) Quantitative analysis of immunostaining results. ∗*P* < 0.05, ∗∗*P* < 0.01, ∗∗∗*P* < 0.001.

Next, immunohistochemical staining was performed to assess the changes in the expression of proteins associated with salivary secretion and immune-inflammatory processes in the submandibular gland tissues across experimental groups. As shown in (Fig. 7 and S8), compared with healthy mice, xerostomia mice exhibited significantly reduced expression levels of AQP5 (aquaporin 5) and AMY1 (α-amylase), alongside marked upregulation of MUC1 (mucin 1), IL-1β and TNF-α. AQP5, a pivotal aquaporin in salivary glands, primarily regulates cellular water homeostasis, facilitating saliva production and secretion to maintain oral moisture [31].AMY1, the major digestive enzyme in saliva, is crucial for the initial digestion of starch, and its expression level serves as a functional indicator of salivary gland activity [32].MUC1, an essential mucin, often shows abnormal expression during inflammation, closely linked to excessive mucus secretion and the amplification of inflammatory cascades [33]. IL-1β and TNF-α are key pro-inflammatory cytokines that orchestrate the inflammatory response by inducing the release of various inflammatory mediators, thereby exacerbating tissue inflammation [34].Following intravenous administration of Ce@gPEI, significant upregulation of AQP5 and AMY1 expression was observed, accompanied by a notable downregulation of MUC1, IL-1β and TNF-α expression, with these changes achieving statistical significance. These results indicate that Ce@gPEI effectively modulates the expression of key functional proteins in submandibular gland tissue, thereby inhibiting inflammatory responses and alleviating xerostomia symptoms.

**Fig. 8:**
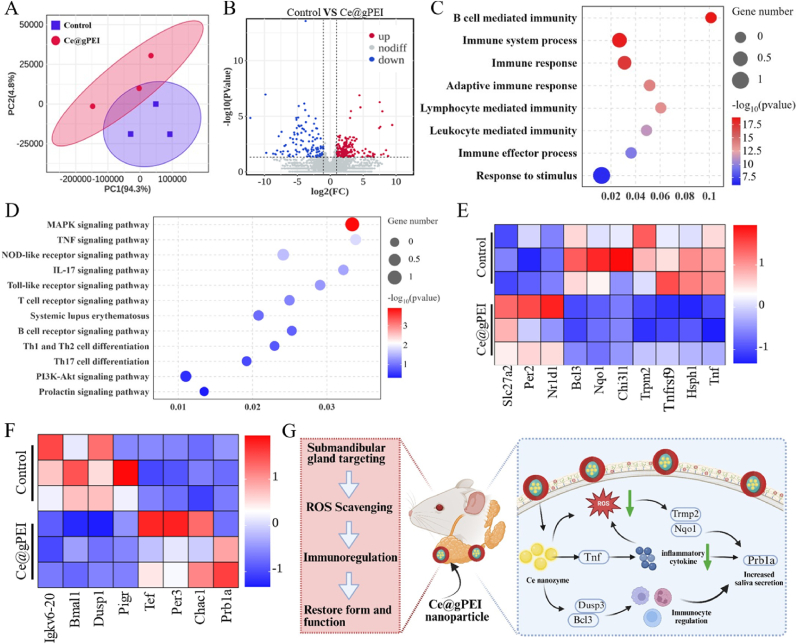
**Transcriptomic analysis of submandibular gland tissues in xerostomia mice following Ce@gPEI treatment.** (A) PCA showing the separation of gene expression profiles between the Ce@gPEI-treated and xerostomia groups. (B) Volcano plot illustrating DEG between Ce@gPEI-treated and xerostomia groups. (C, D) GO enrichment analysis (C) and KEGG pathway enrichment analysis (D) of DEGs in the Ce@gPEI-treated versus xerostomia group. (E) Heatmap of anti-inflammatory and antioxidant-related genes. (F) Heatmap of genes related to tissue repair and saliva secretion. (G) Diagram of engineering nanoenzyme targeting submandibular gland for the treatment of xerostomia.

To gain deeper insights into the underlying mechanisms through which Ce@gPEI ameliorates xerostomia, RNA sequencing (RNA-seq) analysis was conducted on the submandibular gland tissues. Principal component analysis (PCA) results revealed a distinct separation between the Ce@gPEI treatment group and the control group along the primary variation direction (PC1), suggesting that Ce@gPEI exerts a significant impact on salivary gland function and inflammatory responses. In contrast, minimal differences were observed between the two groups along the secondary component axis (PC2) (Fig. 8A). Comparative analysis identified 123 downregulated genes and 179 upregulated genes (*P* < 0.05) in the Ce@gPEI treatment group relative to the control group (Fig. 8B).

Gene Ontology (GO) enrichment analysis revealed that Ce@gPEI treatment was intricately associated with multiple biological processes, including B cell-mediated immune response, adaptive immune response, lymphocyte-mediated immune response, and response to stimulus (Fig. 8C). Further, KEGG pathway enrichment analysis showed that Ce@gPEI significantly modulated several immune-related signaling pathways, such as NOD-like receptor signaling pathway, IL-17 signaling pathway and Th1/Th2 cell differentiation pathway. Notably, the TNF signaling pathway was markedly downregulated in the Ce@gPEI treatment group, while the salivary secretion pathway was significantly upregulated (Fig. 8D). These findings suggest that Ce@gPEI alleviates the disease state in mice and promotes salivary secretion by precisely regulating the immune response and reducing the expression of pro-inflammatory factors. At the gene level, Ce@gPEI upregulated immunoregulatory genes (e.g., *Bcl3*) while downregulating pro-inflammatory genes (e.g., *Tnf*, *Chi3l1*). The downregulation of the antioxidant gene *Nqo1* suggests a reduced demand for endogenous defenses due to the potent ROS-scavenging activity of Ce@gPEI (Fig. 8E). A heatmap analysis confirmed the downregulation of ROS-producing genes and the upregulation of genes involved in tissue repair and salivary secretion (e.g., *Prb1a*; Fig. 8F). These findings collectively indicate that Ce@gPEI acts through immunomodulation, redox balance restoration, and functional pathway activation (Fig. 8G).

To validate our transcriptomic findings at the histological, cellular, and protein levels, we performed a comprehensive analysis of the spleen, a key secondary lymphoid organ. Immunohistochemical examination of submandibular gland sections revealed a marked resolution of immune cell infiltration and inflammatory lesions in Ce@gPEI-treated mice compared to the saline control (Figure S10A-B). Consistent with this histological improvement and the downregulation of B cell-mediated immunity pathways from RNA-seq, flow cytometric analysis demonstrated that Ce@gPEI treatment significantly reduced the frequencies of germinal center B (GCB) cells and plasmablasts (PBs) in both the spleen and submandibular glands (Figure S9A-H). Furthermore, this multi-faceted suppression of pathogenic B-cell response was underpinned by a significant decrease in the serum level of B-cell activating factor (BAFF), a pivotal cytokine for B-cell survival and differentiation (Figure S10C). Collectively, these results from solid organ to serum provide corroborating evidence that our nanozyme immunomodulator effectively alleviates the systemic aberrant B-cell response, a cornerstone of pSS pathogenesis.

## 3 Discussion and conclusion

In this study, we propose a novel engineered nanozyme immunomodulator, Ce@gPEI, which effectively alleviates xerostomia in a murine model by regulating the submandibular gland and restoring its immune and functional homeostasis. Our findings underscore the potential of combining targeted nano-delivery with multi-enzyme mimetic activity as a sophisticated strategy for treating salivary gland dysfunction and its symptomatic manifestation, xerostomia. A pivotal aspect of our design was achieving specific accumulation within the salivary gland. While acetylcholine receptors are widely distributed, our in vivo imaging and M3R blockade experiments confirmed that Ce@gPEI preferentially accumulates in the submandibular gland. This observed selectivity is likely attributable to the combined effect of the reported affinity of surface-exposed PEI segments for the densely expressed M3 muscarinic receptor, a key regulator of salivary secretion, and the unique physiological microenvironment of the target tissue [23]. We acknowledge that systemic administration also leads to significant sequestration by the reticuloendothelial system (RES), as evidenced by accumulation in the liver and spleen. This is a common challenge for nanotherapeutics and presents a future opportunity to further enhance targeting efficiency through surface engineering.

Once accumulated in the submandibular gland, Ce@gPEI exerts its therapeutic efficacy through its potent ROS-scavenging capabilities, mimicking the activities of catalase (CAT), hydroxyl radical scavenging, and superoxide dismutase (SOD). By mitigating oxidative stress, a well-established driver of glandular damage, the nanozyme directly counteracts a core pathological mechanism in hyposalivation. This antioxidant action was complemented by a profound immunomodulatory effect, observed both systemically and, more importantly, within the local glandular microenvironment. Our flow cytometric analysis of salivary gland tissues revealed a significant shift towards an anti-inflammatory state, characterized by an increase in M2 macrophages and regulatory T cells (Tregs). This local rebalancing of immune cell populations, moving away from a pro-inflammatory (M1/Th1) and towards a reparative (M2/Th2/Treg) phenotype, is crucial for resolving chronic inflammation and facilitating tissue repair.

The transcriptomic data provide a systematic view of Ce@gPEI-induced immunomodulation. The treatment significantly downregulated key pro-inflammatory pathways, including TNF and IL-17 signaling. Notably, the observed modulation of B cell-mediated immunity and the significant reduction in SSA/SSB autoantibodies are of particular relevance to Sjögren's Disease (SjD) where B cell hyperactivity is a hallmark of pathogenesis. This suggests that our therapeutic strategy may be especially pertinent for autoimmune-driven hyposalivation. Concurrently, the upregulation of the salivary secretion pathway and key functional proteins functional proteins (AQP5, AMY1) underscores a molecular-level restoration of glandular function, addressing a core objective in managing xerostomia irrespective of its etiology. When contextualizing our work within the broader field of xerostomia therapeutics, current options primarily offer symptomatic relief or non-specifically suppress the immune system. Our Ce@gPEI platform represents a paradigm shift by simultaneously addressing oxidative stress and immune dysregulation directly at the site of pathology. This is particularly promising for complex conditions like SjD, where both innate and adaptive immune responses are involved. This targeted, multi-mechanistic approach not only enhances therapeutic efficacy but also minimizes the potential for off-target effects, as suggested by the absence of systemic toxicity in our study.

In conclusion, the Ce@gPEI nanosystem establishes a novel therapeutic modality for hyposalivation and salivary gland injury. By integrating targeted delivery, potent antioxidant defense, and multi-faceted immunomodulation, it successfully halts disease progression and promotes functional recovery. Its impact on key SjD-related immune markers further highlights its potential applicability in this challenging autoimmune context. Future work will focus on optimizing the formulation to reduce RES uptake, exploring the long-term biodistribution and safety profile, and validating its efficacy in more complex disease models, with the ultimate goal of translating this promising nanomedicine to clinical application.

## 4 Methods

### 4.1 Materials

The A-B-A type triblock copolymer PEI-PLGA, with a PEI molecular weight of 25,000 (primary, secondary, and tertiary amine ratios of 43.4) and a PLGA molecular weight of 36,000 Da (comprising D, L-lactic acid and glycolic acid in a 75:25 M ratio), was sourced from Olyue Biology (Xian, China). Fluorescent dyes Rhodamine B were purchased from Sigma-Aldrich (St. Louis, USA), respectively. Cerium nitrate (Ce(NO_3_)_3_·6H_2_O) was obtained from Aladdin; Ammonia (NH_3_·H_2_O, 30 %) was purchased from Aladdin. Hydrogen peroxide (H_2_O_2_, 30 %) was obtained from Sigma; 2,7-dichlorofluorescein diacetate (DCFH-DA) was obtained from Solarbio Co., Ltd.

### 4.2 Cell culture

In this study, we utilized the murine macrophage line RAW264.7 and human umbilical vein endothelial cells (HUVECs) to evaluate Ce@gPEI bioactivity. Cells were maintained in Dulbecco's Modified Eagle Medium (DMEM) containing 10 % fetal bovine serum and 1 % penicillin-streptomycin antibiotic mixture. Cells were maintained in a humidified incubator at 37 °C with 5 % CO_2_. The culture medium was refreshed every two days. Subculturing was performed when cells reached 80–90 % confluence using 0.25 % trypsin-EDTA solution, following a standard split ratio of 1:3 to 1:4 for routine passage. For all experiments, cells in the logarithmic growth phase were used.

### 4.3 Synthesis of Ce nanozyme

Cerium oxide nanozymes (CeNPs) were synthesized through a co-precipitation method. Briefly, 217 mg of cerium nitrate hexahydrate dissolved in 4 mL of deionized water was mixed with 200 mg of citric acid in 2 mL of water. This precursor solution was rapidly introduced into 100 mL of a 0.4 M ammonium hydroxide solution under constant stirring. The reaction was allowed to proceed for 24 h at room temperature, yielding a transparent yellow colloidal suspension. To remove large aggregates, the crude product was first centrifuged at 2600×*g* for 30 min. The collected supernatant was then concentrated and purified using a 3 kDa molecular weight cut-off centrifugal filter. For further refinement, the nanosuspension underwent ultracentrifugation at 15,000×*g* for 10 min, and the final product was sterilized by passing through a 0.02 μm filter. The purified CeNPs were obtained as a powder through lyophilization and stored at 4 °C for future use.

### 4.4 Ce@gPEI nanocomposites synthesis

The core-shell Ce@gPEI nanocomposites were assembled using a water-in-oil-in-water (W/O/W) double emulsion approach. In a typical procedure, 20 mg of the PEI-PLGA copolymer was first dissolved in 1 mL of dichloromethane to form the organic phase. An inner aqueous phase (0.2 mL), containing either deionized water for blank particles, Rhodamine B for fluorescent labeling, or a suspension of pre-synthesized CeNPs for Ce@gPEI, was then added to the polymer solution. This two-phase mixture was emulsified by sonication at 35 W for 5 min, forming the primary W/O emulsion. Next, this primary emulsion was poured into 2 mL of an aqueous 2 % polyvinyl alcohol (PVA) solution and subjected to a second round of sonication for 5 min to create the stable W/O/W double emulsion. The resulting emulsion was transferred into a 0.6 % PVA solution under continuous stirring, and dichloromethane was thoroughly eliminated by rotary evaporation, leading to the formation of solidified nanoparticles.

### 4.5 Characterization of nanoparticles

A comprehensive set of techniques was employed to characterize the physicochemical properties of the synthesized nanomaterials. Transmission electron microscopy (TEM) was used to assess the general morphology and size of the nanoparticles. The crystalline structure and lattice fringes of individual CeNPs were resolved using high-resolution TEM (HR-TEM). The surface elemental composition and the valence states of cerium (Ce^3+^ and Ce^4+^) were analyzed by X-ray photoelectron spectroscopy (XPS) on a Physical Electronics PHI 5700 system. In aqueous suspension, the hydrodynamic diameter distribution and surface charge (zeta potential) were determined by dynamic light scattering (DLS). To quantify the polymeric content within the composite nanozymes, thermogravimetric analysis (TGA) was performed under an air atmosphere.

### 4.6 Enzyme activity assay of Ce nanozyme

The multi-enzyme mimetic activities of the Ce nanozymes, including superoxide dismutase (SOD), catalase (CAT), and hydroxyl radical (•OH) scavenging capabilities, were comprehensively evaluated with corresponding commercial assay kits. For the SOD-like activity assessment, CeNPs were serially diluted in PBS, and 10 μL of each concentration was dispensed into a 96-well plate. The reaction was then initiated by adding the kit's substrate and xanthine oxidase components according to the manufacturer's guidelines, followed by a 20-min incubation at room temperature prior to measuring the absorbance at 450 nm. The CAT-mimetic activity was tested using a hydrogen peroxide/peroxidase-based system. Briefly, 50 μL of gradient CeNP solutions, prepared in the specified reaction buffer, were combined with an equal volume of 40 μM H_2_O_2_ in the wells. Following a 20-min reaction period, 50 μL of the developer working solution was added, and the mixture was incubated for an additional 30 min before the absorbance was read at 560 nm. Finally, the •OH scavenging capacity was quantified using a specialized Hydroxyl Radical Scavenging Assay Kit, with all procedures meticulously performed as directed by the instructions.

### 4.7 Cellular biocompatibility

The cytotoxicity of the multifunctional nanoparticles was evaluated on RAW264.7 macrophages and HUVEC. Cells were seeded into a 24-well plate at a density of 1 × 10^5^ cells per well. Following an overnight incubation, the culture medium was replaced with fresh medium containing varying concentrations of the nanoparticles, and the cells were incubated for an additional 24 h. Cell viability was subsequently assessed using Live/Dead staining, following the protocol provided by the ThermoFisher Live/Dead staining kit. For cell proliferation studies, cells were plated in a 96-well plate, cultured overnight, and then exposed to different concentrations of nanoparticles for 24 h. The CCK8 assay was conducted in accordance with the instructions from the Solarbio CCK8 kit, with absorbance measured at 450 nm using a microplate reader.

### 4.8 Determination of intracellular ROS scavenging

To investigate the capacity of Ce@gPEI nanozymes to eliminate intracellular reactive oxygen species (ROS), hydrogen peroxide (H_2_O_2_) was employed to mimic ROS overproduction conditions. Initially, the optimal concentration of H_2_O_2_ for stimulation was determined. RAW264.7 macrophages were seeded in 96-well plates at a density of 5 × 10^3^ cells per well and allowed to adhere overnight. The cells were then stimulated with a gradient of H_2_O_2_ concentrations (0, 100, 200, 300, 600, 800, and 1000 μM) in fresh medium for 2 h at 37 °C. After replacing the H_2_O_2_-containing medium with fresh complete medium, the cells were further incubated for 24 h. Cell viability was subsequently measured using the CCK-8 assay.

For the cytoprotection experiment, RAW264.7 cells were seeded as described above. Each well received 100 μL of fresh medium containing 600 μM H_2_O_2_ and was incubated at 37 °C for 2 h. The medium was then discarded, and the wells were rinsed once with phosphate-buffered saline (PBS). Subsequently, each well received 100 μL of complete medium containing Ce@gPEI nanozymes at varying concentrations (0, 5, and 10 μg/mL), followed by 24 h of co-incubation, with three replicates per concentration. The subsequent steps followed the cytotoxicity test protocol described earlier. A normal control group (without H_2_O_2_ stimulation) was also included. The same experiment was also repeated with HUVECs.

#### 4.8.1 ROS clearance ability

RAW264.7 macrophages were seeded in 6-well plates at a density of 2 × 10^6^ cells per well and incubated with Ce@gPEI nanozymes at various concentrations for 24 h. Following this pretreatment, cells were incubated with 10 μM 2,7-dichlorofluorescein diacetate (DCFH-DA) in serum-free medium at 37 °C for 20 min in the dark. Subsequently, the cells were rinsed twice with serum-free medium to remove excess probe and then exposed to 600 μM H_2_O_2_ in complete medium for 2 h at 37 °C to induce ROS generation.

For flow cytometric analysis, the cells were immediately harvested by gentle scraping into cold PBS, centrifuged at 300×*g* for 5 min, and resuspended in PBS supplemented with 1 % FBS. Intracellular ROS levels, as indicated by DCF fluorescence, were quantified using a BD FACSCalibur flow cytometer with excitation at 488 nm and emission detection at 525 nm (FL1 channel). A total of 10,000 events were collected per sample. Data analysis was performed using FlowJo software (version 10.8.1). For comparison, RAW264.7 macrophages untreated with H_2_O_2_ served as the negative control group, while those treated with H_2_O_2_ but not pretreated with the nanozymes were designated as the model (positive control) group.

#### 4.8.2 ELISA

Cell culture supernatants and mouse serum samples were harvested and centrifuged at 1000×*g* for 10 min to remove particulates. The concentrations of SSA antibodies, SSB antibodies, TNF-α, IL-1β, IL-10, IL-6, and BAFF were determined using commercial ELISA kits (Jingmei Biotechnology, Jiangsu, China) in strict accordance with the manufacturer's guidelines. Briefly, 100 μL of standards or samples were added to the antibody-precoated wells and incubated at 37 °C for 90 min. After washing five times with Wash Buffer, 100 μL of biotinylated detection antibody was added to each well and incubated at 37 °C for 60 min. Following another washing step, 100 μL of Horseradish Peroxidase (HRP)-conjugated streptavidin was added and incubated at 37 °C for 30 min protected from light. After a final wash, 100 μL of Tetramethylbenzidine (TMB) substrate was added and incubated at 37 °C for 15 min for color development. The reaction was terminated by adding 50 μL of Stop Solution, and the absorbance was immediately measured at 450 nm using a microplate reader (BioTek Instruments, USA). A standard curve was generated for each assay, and sample concentrations were calculated by interpolation. All samples were assayed in duplicate.

#### 4.8.3 Animal experiment

The study was approved by the Ethics Committee of Nantong University School of Medicine. All animal procedures were conducted in accordance with the institutional guidelines for the care and use of laboratory animals. 11-week-old female NOD/ShiLtJ mice with spontaneous xerostomia were randomly divided into four experimental groups (n = 6 per group): (1) control group (administered saline); (2) PEI group; (3) CeNP group; and (4) Ce@gPEI group. Age- and sex-matched C57BL/6J (B6) mice (n = 6) served as the healthy control group. All mice were housed under specific pathogen-free conditions with a 12 h light/dark cycle and provided ad libitum access to food and water. Each mouse received a weekly intravenous injection via the tail vein of 100 μL containing the respective nanoparticles (PEI, CeNP, or Ce@gPEI at a dosage equivalent to 30 mg/kg for Ce@gPEI) or an equal volume of sterile saline for the control groups. The treatment period lasted for 8 consecutive weeks. Body weight and general health status were monitored weekly throughout the study. After 8 weeks of treatment, all mice were humanely euthanized by CO_2_ inhalation, and tissues (including submandibular glands, spleen, and other major organs) were collected for subsequent analysis.

#### 4.8.4 Determination of salivary flow rate in mice

Salivary flow rate was measured prior to euthanasia using a commercial saliva secretion test strip (registered with the Tianjin Medical Device Administration, No. 20172200300) to improve accuracy and minimize tissue damage associated with traditional methods. The procedure was conducted as follows: each mouse received an intraperitoneal injection of pilocarpine hydrochloride (2 mg/kg body weight) to stimulate salivary secretion. After a 5-min rest period, a standardized test strip was placed under the mouse's mouth for exactly 5 min to collect saliva. The length of the saliva column formed on the strip was measured in centimeters. The salivary flow rate was calculated using the formula: Salivary Flow Rate (cm/min) = Length of Saliva Column (cm)/Collection Time (min). The obtained values were then normalized to the animal's body weight (g) to account for individual size differences. All measurements were performed under consistent conditions to ensure reproducibility.

#### 4.8.5 Histological analysis

Following euthanasia, major organs—including the submandibular gland, spleen, kidney, lung, liver, and pancreas—were promptly collected. Organ morphology was documented, and the weights of the spleen and submandibular gland were recorded. Tissues were fixed in 10 % neutral buffered formalin for 24 h at room temperature, followed by standard dehydration, paraffin embedding, and sectioning at 5 μm thickness. Hematoxylin and eosin (H&E) staining was performed to evaluate general histoarchitecture and inflammatory infiltration.

Acinar atrophy in the submandibular gland was assessed using a semi-quantitative scoring system based on H&E-stained sections. Two blinded pathologists independently examined five non-overlapping fields per sample at × 200 magnification and scored them as follows: 0 (normal), 1 (mild atrophy, minimal acinar size reduction), 2 (moderate atrophy with mild disorganization), 3 (severe atrophy with prominent disorganization), and 4 (very severe atrophy with extensive fibrosis and near-total acinar loss). Inter-observer agreement was confirmed (Cohen's kappa >0.8), and discrepancies were resolved by consensus. The mean score per sample was used for statistical analysis.

To assess tissue fibrosis, Masson's trichrome staining and Sirius red staining were performed on submandibular gland sections according to standard protocols, highlighting collagen fibers in blue and green/red, respectively. All stained sections were evaluated under a light microscope (Olympus, Japan) by two blinded observers. This.

### 4.9 Flow cytometry analysis

Single-cell suspensions were prepared from spleen and submandibular gland tissues. Splenic tissues were mechanically dissociated through a 70-μm cell strainer. Submandibular glands were minced and subjected to enzymatic digestion using a cocktail of 1 mg/mL collagenase IV, 0.5 mg/mL hyaluronidase, and 20 μg/mL DNase I in RPMI-1640 supplemented with 2 % FBS for 30 min at 37 °C with continuous agitation. Following digestion, cells were filtered through a 70-μm strainer, washed, and resuspended in FACS buffer (PBS containing 1 % BSA) for subsequent staining.

To prevent nonspecific antibody binding, cells were first treated with Fc Block (anti-CD16/32) on ice for 15 min. Surface staining was then performed by incubating the cells with titrated antibody cocktails for 30 min at 4 °C in the dark. For intracellular cytokine detection (IFN-γ, IL-4, IL-17A), a 5-h stimulation was conducted using PMA (50 ng/mL) and ionomycin (1 μg/mL) in the presence of GolgiStop. Subsequently, the cells were fixed and permeabilized following the protocol of the Cyto-Fast™ Fix/Perm Buffer Set. For transcription factor staining (FOXP3), intracellular detection was performed using the Foxp3/Transcription Factor Staining Buffer Set according to the manufacturer's protocol.

Comprehensive antibody information is listed as follows:

T helper cell panel (TH1/TH2/TH17): Fixable Viability Stain 455UV; CD3-APC-Cy7 (BD Pharmingen, 557596); CD4-FITC (BD Pharmingen, 553046); IFN-γ-PE (BD Pharmingen, 554412); IL-4-APC (BD Pharmingen, 554436); IL-17A-BV421 (BD Pharmingen, 563354).

Treg panel: Fixable Viability Stain 455UV; CD3-APC-Cy7 (BD Pharmingen, 557596); CD4-FITC (BD Pharmingen, 553046); CD25-PE (BD Pharmingen, 561065); FOXP3-BV421 (Biolegend, 126419); ICOS-BV605 (BD Pharmingen, 745254); PD-1-APC (BD Pharmingen, 562671); CXCR5-PE-Cy7 (eBioscience™, 25-7185-80).

Germinal center and B cell panel (GCB): Fixable Viability Stain 455UV; TCRβ-PerCP-Cy5.5 (eBioscience™, 45-5961-82); B220-APC (BD Pharmingen, 561880); CD19-BV510 (BD Pharmingen, 562956); CD69-PE-Cy5 (Biolegend, 104509); GL-7-PE (Biolegend, 144607); FAS-APC-R700 (BD Pharmingen, 752226); CD38-PE-CF594 (Biolegend, 102729).

Plasmablast and B cell subset panel (PB): Fixable Viability Stain 455UV; TCRβ-PerCP-Cy5.5 (eBioscience™, 45-5961-82); B220-APC (BD Pharmingen, 561880); CD19-BV510 (BD Pharmingen, 562956); CD138-BV605 (Biolegend, 142515); CD5-APC-R700 (Biolegend, 100635); CD1d-PE (BD Pharmingen, 553846); IgA-BV650 (BD Pharmingen, 743296); IgM-APC-Cy7 (Biolegend, 406515); IgG-BV421 (BD Pharmingen, 553969); IgD-FITC (BD Pharmingen, 564273).

Stained cells were resuspended in FACS buffer and acquired on a BD FACSCanto II flow cytometer. A minimum of 10,000 events per target population were collected. Data analysis was performed using FlowJo software (v10.8.1), with gating strategies established based on fluorescence-minus-one (FMO) controls and isotype-matched antibodies.

#### 4.9.1 Data analysis

Prism8.0 software was used for data analysis and statistics, and one-way ANOVA was employed to examine group differences. When *p* < 0.05, a difference was deemed statistically significant.

## CRediT authorship contribution statement

**Xinyu Tao:** Writing – original draft, Methodology, Formal analysis, Data curation. **Rui Zhao:** Supervision, Software. **Ye Fang:** Methodology, Investigation. **Minjie Chen:** Project administration, Investigation. **Cong Xu:** Supervision, Methodology. **Yujuan Zhu:** Conceptualization. **Zhifeng Gu:** Conceptualization.

## Declaration of competing interest

The authors declare that they have no known competing interests or personal relationships that could have appeared to influence the work reported in this paper.

## Data Availability

Data will be made available on request.
