# Supplementary material for: Engineered nanozyme immunomodulator for xerostomia treatment via regulating submandibular salivary gland
Source: Mater Today Bio. 2025 Nov 13;35:102555. doi: 10.1016/j.mtbio.2025.102555 (PMC12664455; doi:10.1016/j.mtbio.2025.102555)
Supplement: Multimedia component 1 [file mmc1.docx]

**Supplementary Information**

**Engineered nanozyme immunomodulator for xerostomia treatment via regulating submandibular salivary gland**

Xinyu Tao^1^, Rui Zhao^1^, Ye Fang^1^, Minjie Chen^1^, Cong Xu^2^, Yujuan Zhu^1*^, and Zhifeng Gu^1*^

^1^ Department of Rheumatology, Research Center of Clinical Medicine, Research Center of Immunology, Affiliated Hospital of Nantong University, Nantong University, Nantong 226001, China

^2^ Department of Biomedical Engineering, Columbia University, New York, NY, 10027, USA.

Correspondence: yujuanzhu@ntu.edu.cn (Y. Zhu) and [guzf@ntu.edu.cn](mailto:guzf@ntu.edu.cn) (Z. Gu)

**Methods**

***Ex Vivo* Imaging of PEI−PLGA Nanoparticles**

Free Rhodamine B and Rhodamine B-labeled PEI nanoparticles (RhB-Ce@gPEI) were administered intravenously to BALB/c mice (n=3 per group). To investigate the role of the muscarinic M3 receptor (M3R) in the targeting process, an additional group of mice (n=3) was pre-treated with the M3R antagonist Tiotropium Bromide (1 mg/kg, intraperitoneal injection) 1 hour prior to the administration of RhB-Ce@gPEI. The fluorescence intensities of all formulations (excitation: 552 nm; emission: 588 nm) were normalized using a spectrophotometer prior to injection. Eight hours post-injection, the mice were euthanized, and their submandibular salivary glands and major organs (including the brain, heart, lung, liver, spleen, kidney, stomach, and intestine) were excised and imaged using a fluorescence imaging system.

***Immunohistochemical staining***

For immunohistochemical analysis, submandibular gland tissue sections (5 μm) were deparaffinized in xylene and rehydrated through a graded ethanol series. Antigen retrieval was performed by heating the sections in 0.1 M citrate buffer (pH 6.0) at 95°C for 20 minutes. Endogenous peroxidase activity was blocked by incubation with 3% hydrogen peroxide for 30 minutes at room temperature. Non-specific binding sites were blocked with 10% normal donkey serum for 1 hour at room temperature.

Sections were then incubated overnight at 4°C with the following primary antibodies: anti-AQP5 (abcam, cat# ab305303, rabbit monoclonal, 1:200), anti-AMY1 (Proteintech, cat# 12540-1-AP, rabbit polyclonal, 1:200), anti-MUC1 (Proteintech, cat# 23614-1-AP, rabbit polyclonal, 1:200), anti-TNF-α (Santa Cruz Biotechnology, cat# sc-52746, mouse monoclonal [52B83], 1:300), anti-IL-1β (Proteintech, cat# 16806-1-AP, rabbit polyclonal, 1:200), anti-F4/80 (Proteintech, cat# 29414-1-AP, rabbit polyclonal, 1:200), anti-CD206 (Proteintech, cat# 18704-1-AP, rabbit polyclonal, 1:200), anti-CD19 (Proteintech, cat# 27949-1-AP, rabbit polyclonal, 1:200), anti-CD8a (CST, cat# 98941T, rabbit monoclonal, 1:200), and anti-CD4 (abcam, cat# ab133616, rabbit monoclonal, 1:200).After washing with PBS, sections were incubated with horseradish peroxidase (HRP)-conjugated goat anti-rabbit or goat anti-mouse secondary antibodies (ZSGB-Bio, Beijing, China) for 1 hour at room temperature. Color development was performed using a DAB substrate kit (ZSGB-Bio, Beijing, China) for 3-5 minutes. Sections were counterstained with hematoxylin for 1 minute, dehydrated through a graded ethanol series, cleared in xylene, and mounted with neutral resin.

The expression levels of target proteins were quantified by measuring the mean optical density in five randomly selected fields per section using ImageJ software (version 1.53) with appropriate immunohistochemistry analysis plugins.

***RNAseq***

Transcriptome analysis of submandibular gland tissue was carried out by Gene Denovo Biotechnology Co. (Guangzhou, China). Total RNA extraction was performed using Trizol reagent. The quality of the RNA was evaluated using an Agilent 2100 Bioanalyzer (Agilent Technologies, Palo Alto, CA, USA) and confirmed via RNase-free agarose gel electrophoresis. Bioinformatics analyses including identification of differentially expressed genes (DEGs), Gene Ontology (GO) enrichment, and KEGG pathway analysis were performed using the Gene Denovo online platform ([www.genedenovo.com](http://www.genedenovo.com)).

**Figures
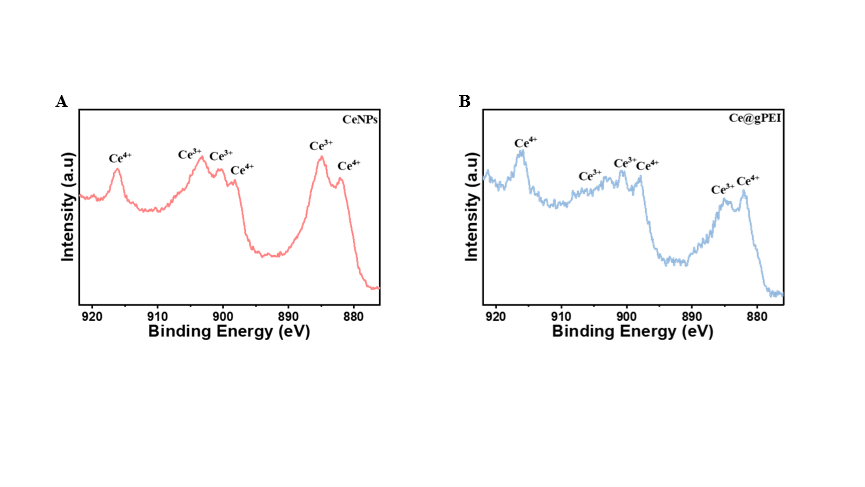
**

**Figure S1.** XPS test result indicating the chemical valence of CeNPs and Ce@gPEI.


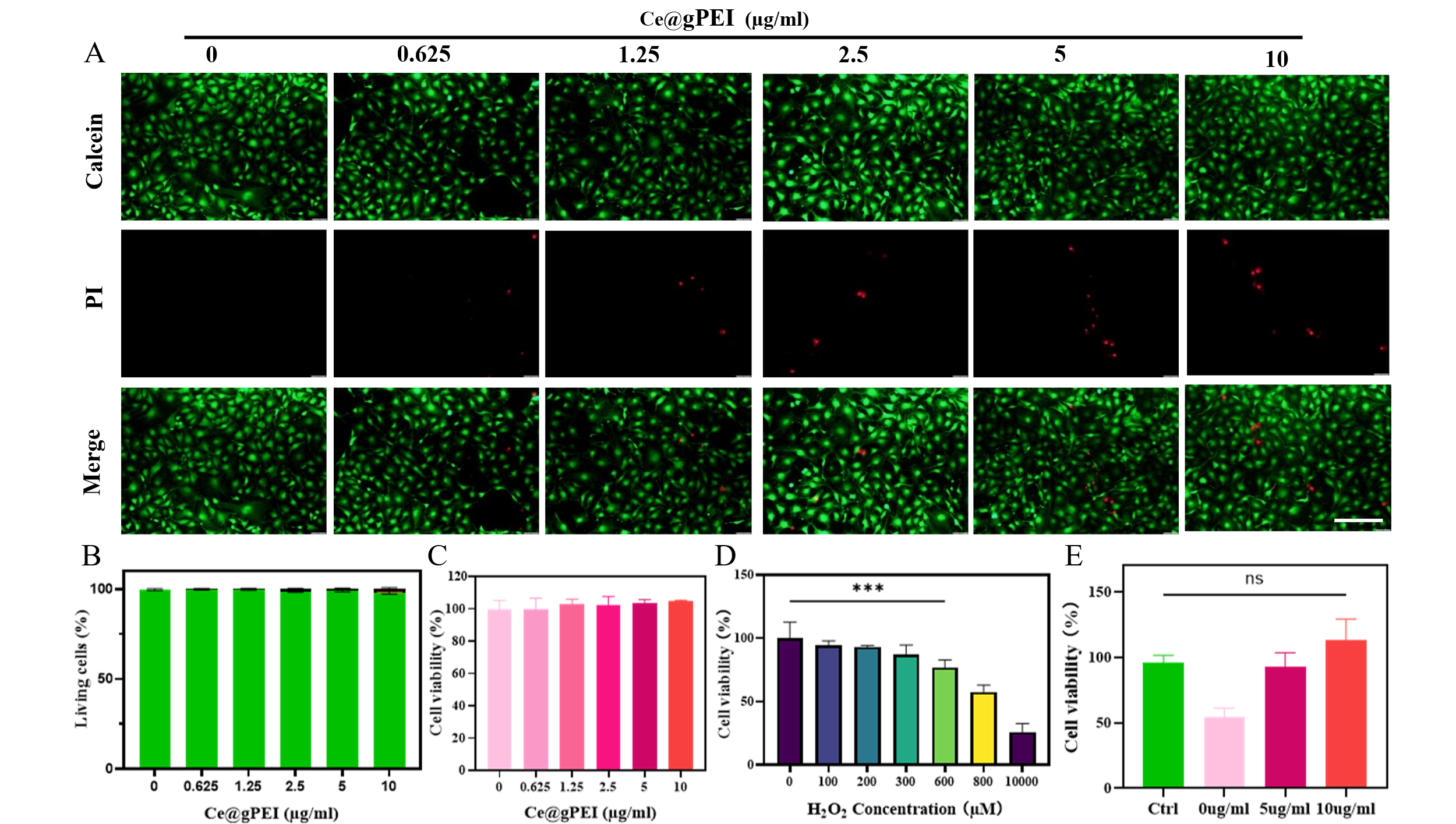


**Figure S2.** (A) Fluorescence images of Calcein/PI staining. Scale bar, 200 μm. (B) Quantitative statistical analysis of viable cells in different groups. (C) Cytotoxicity of Ce@gPEI at various concentrations against HUVECs after 24 h of incubation. (D) Quantitative assessment of HUVEC viability following stimulation with different concentrations of H_2_O_2_. (E) Quantitative viability of HUVECs treated with various concentrations of Ce@gPEI after stimulation with 600 μM H_2_O_2_.

* *P* < 0.05, ** *P* < 0.01, *** *P* < 0.001.


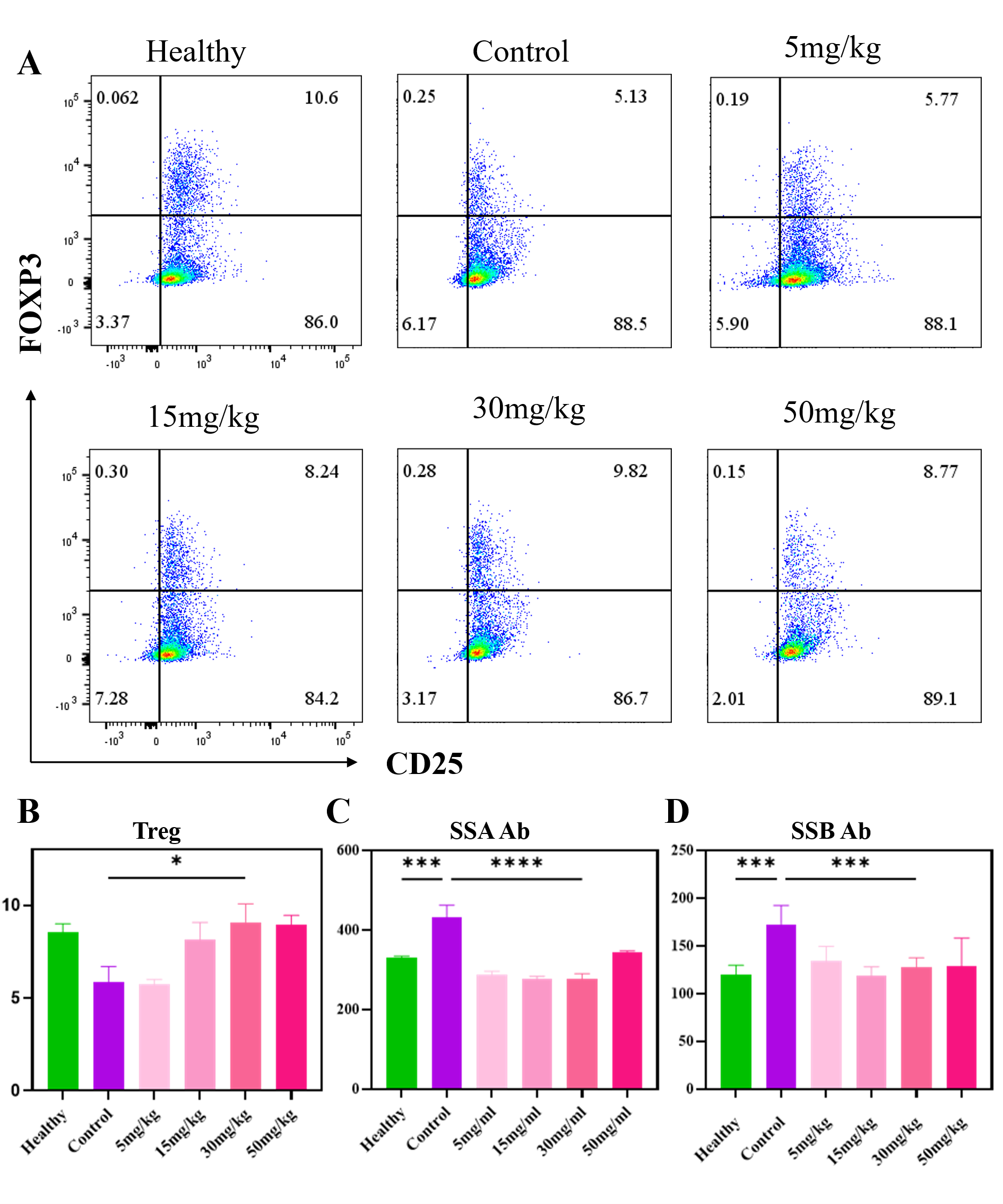
**Figure S3.** (A) Flow cytometry analysis of the proportion of splenic Treg cells in the healthy group, xerostomia group and groups treated with different concentrations of Ce@gPEI. (B) Quantitative analysis of the proportion of Treg cells in different groups via flow cytometry. n = 5, *P* < 0.01. (C-D) ELISA quantification of serum SSA and SSB antibody titers in the healthy group, xerostomia group and groups treated with different concentrations of Ce@gPEI. * *P* < 0.05, ** *P* < 0.01, *** *P* < 0.001.


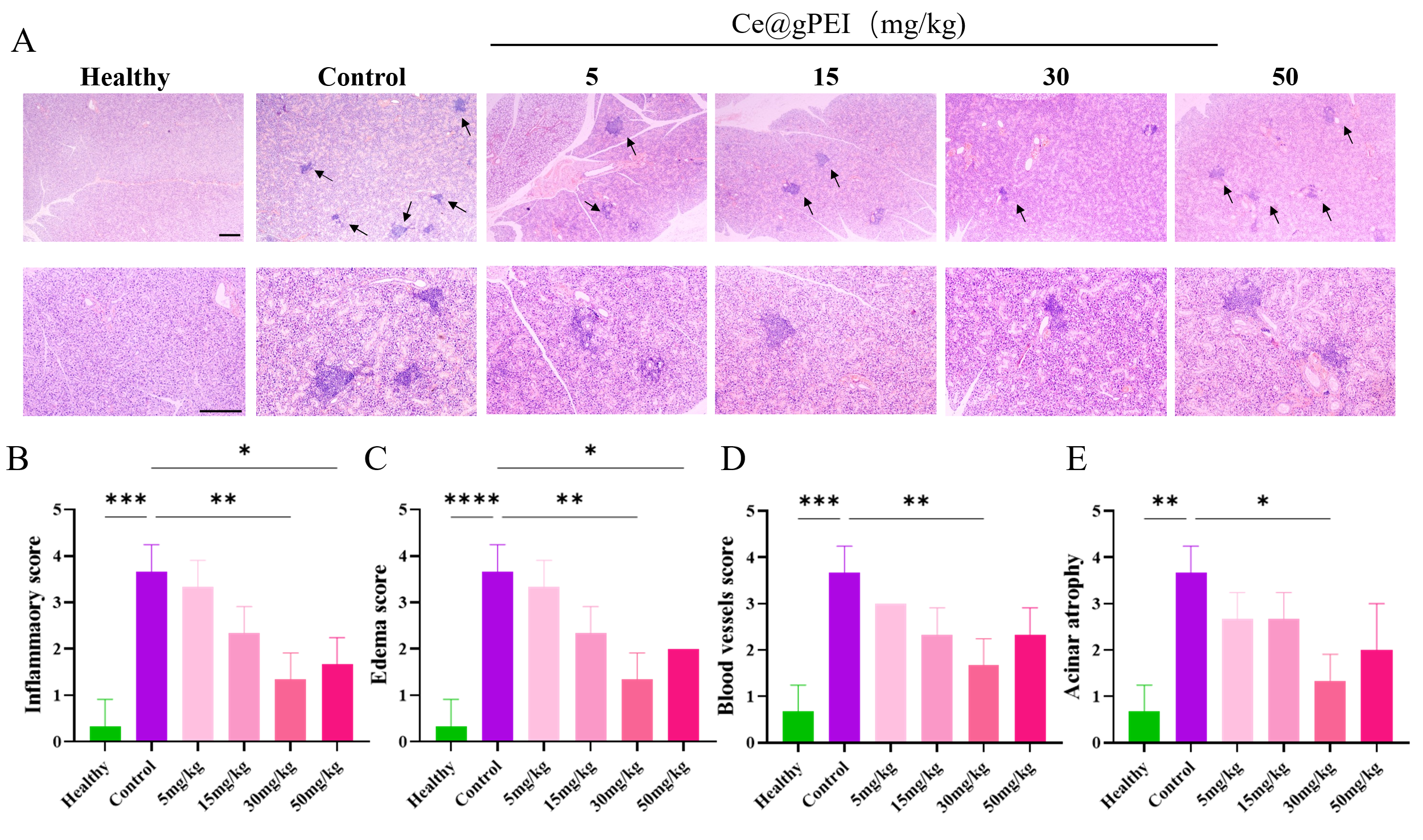
**Figure S4.** (A) HE staining of submandibular gland tissue in different groups. Scale bar, 200 μm. (B-E) Inflammatory infiltration (B), tissue edema (C), vascular congestion (D), acinar atrophy (F) semi-quantitative analysis score in each experimental group. * *P* < 0.05, ** *P* < 0.01, *** *P* < 0.001.


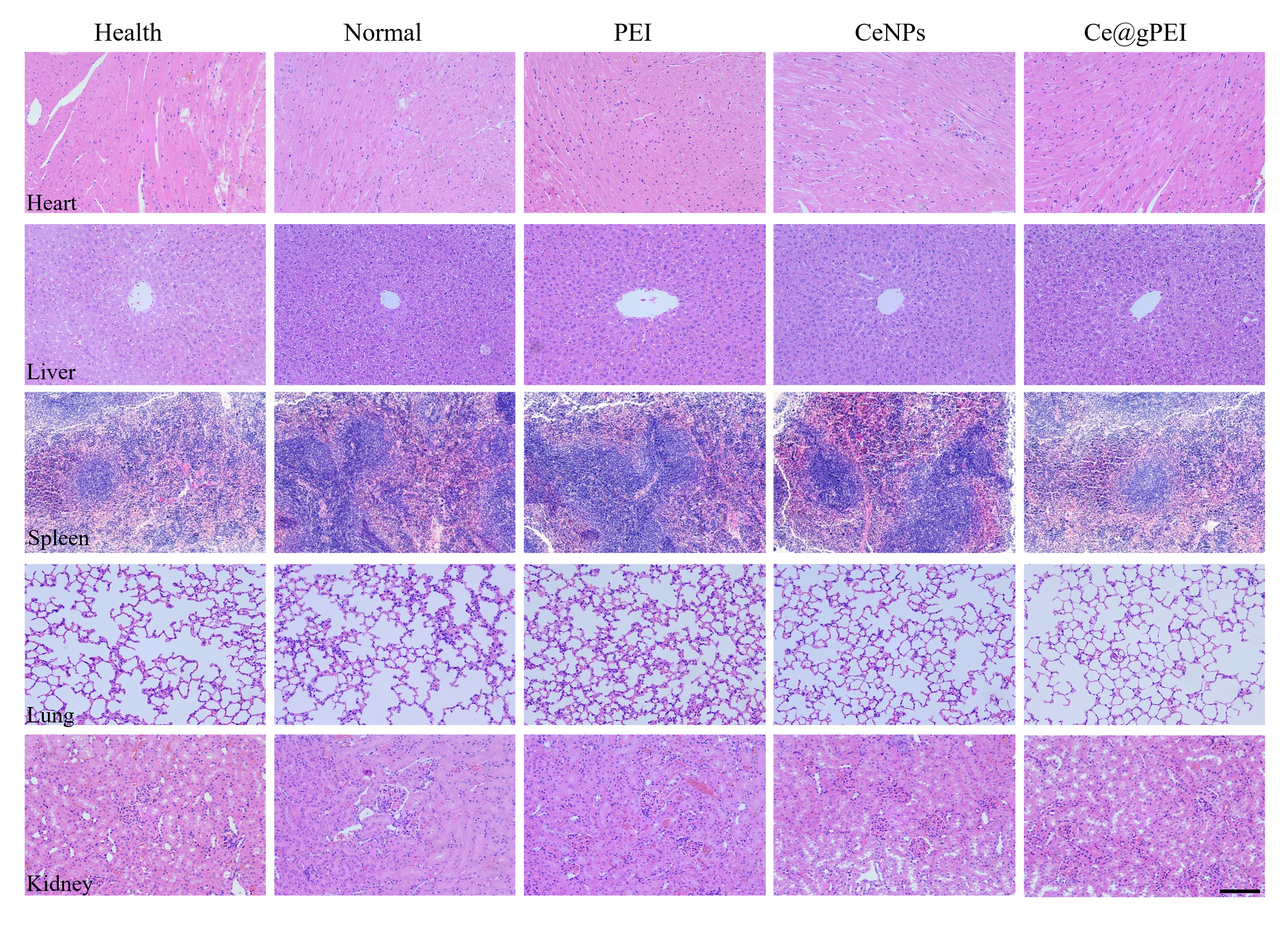


**Figure S5.** HE-staining in each group, including heart, liver, lung, and kidney. Scare bar, 200 μm.

**Figure S6.** Weekly water intake in different experimental groups. 图片居中


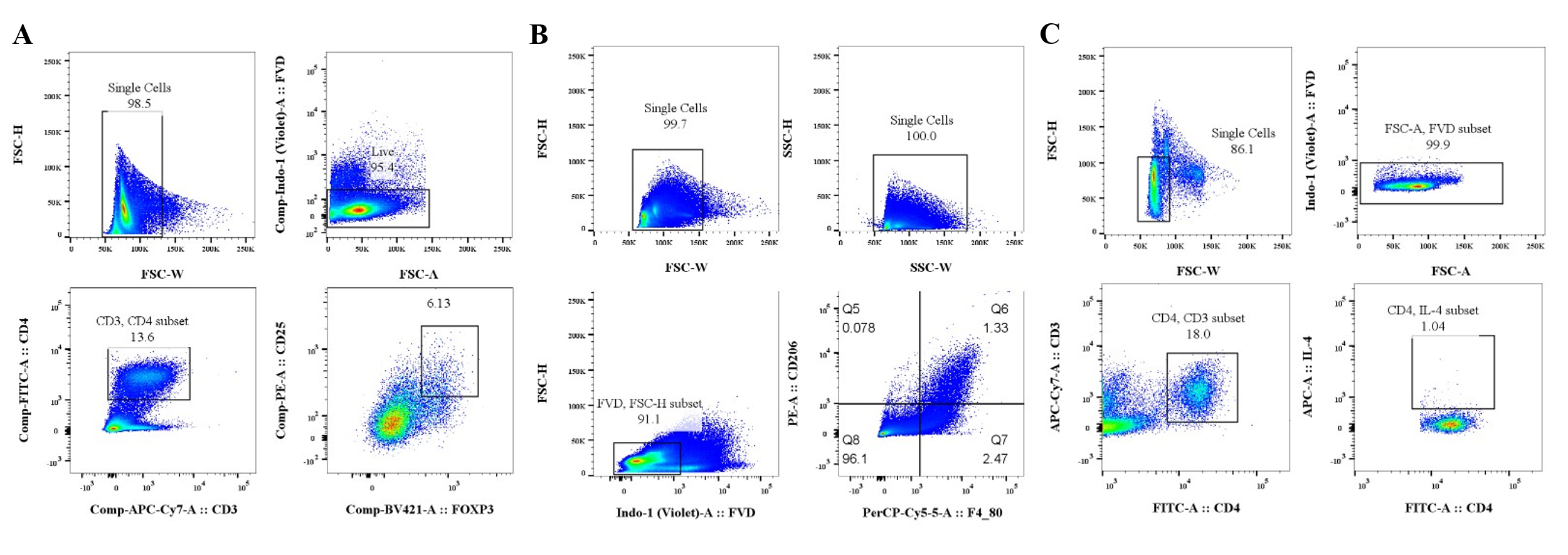


**Figure S7.** Gating strategy for the identification of immune cell populations by flow cytometry. (A) Gating of F4/80⁺CD206⁺ M2 macrophages. (B) Gating of Foxp3⁺CD25⁺ regulatory T cells (Tregs) from the CD4⁺ T cell population. (C) Gating of IFN-γ⁺ Th1 and IL-4⁺ Th2 cells from the CD4⁺ T cell population.


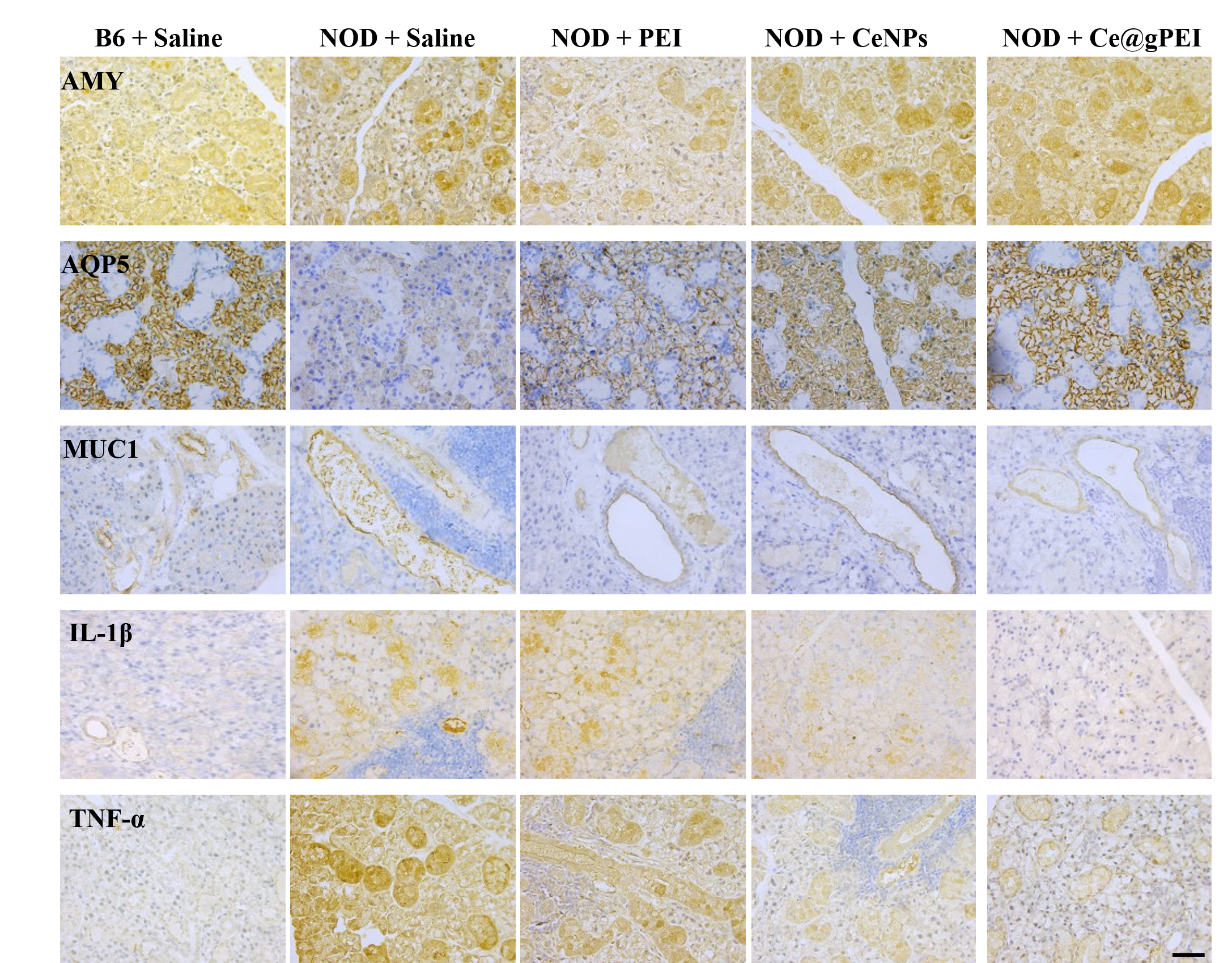
**Figure S8. The pathological change of submandibular gland.** (A) AMY, AQP5, MUC1, IL-1β, and TNF-αimmunohistochemistry in each group. Scare bar, 200 μm.


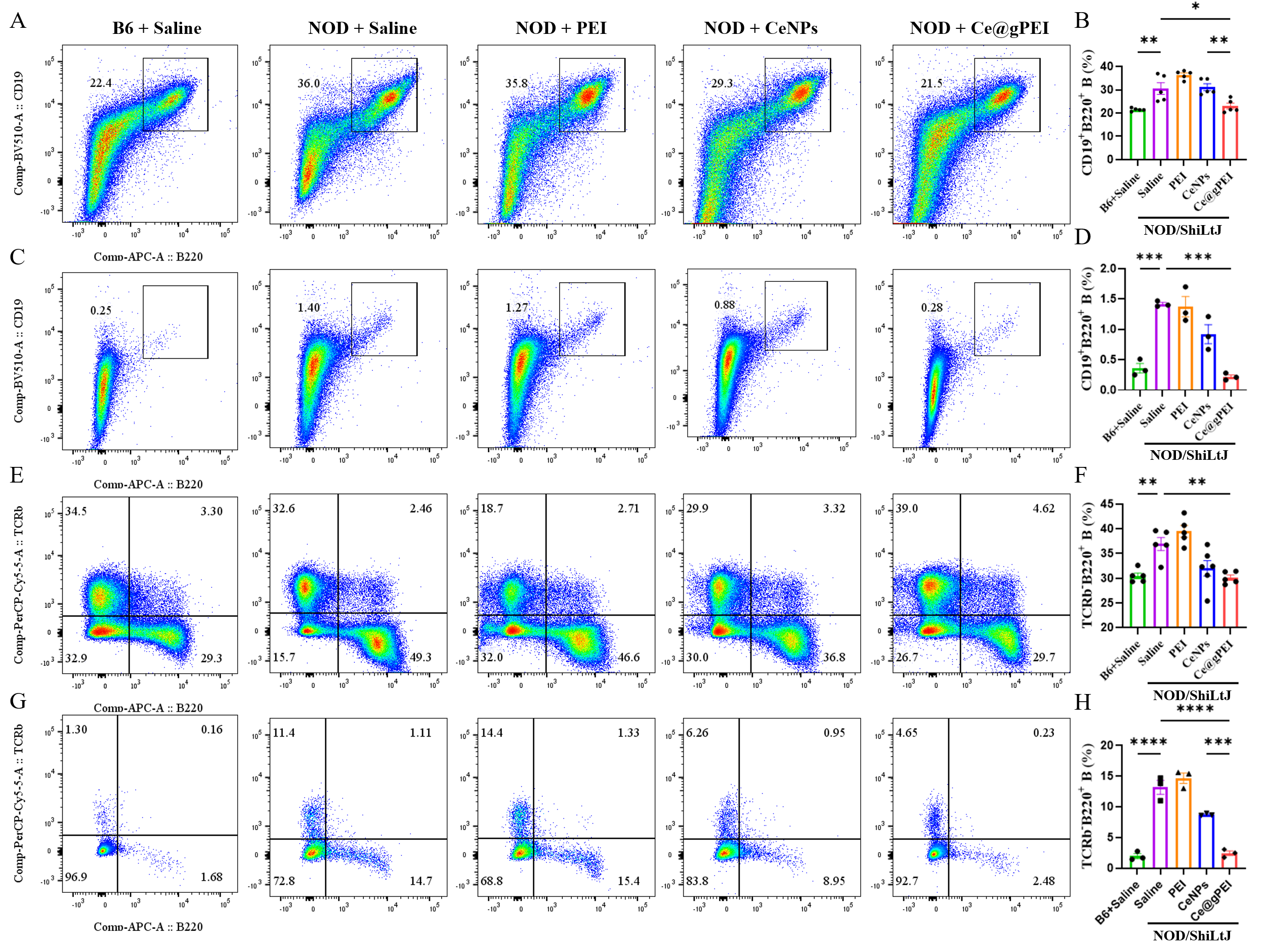


**Figure S9.** Analysis of germinal center B cells (GCB) and plasma blasts (PB) in spleen and submandibular glands across different treatment groups.(A, B) Representative flow cytometry plots (A) and quantitative analysis (B) of GCB cells in the spleen.(C, D) Representative flow cytometry plots (C) and quantitative analysis (D) of GCB cells in the submandibular glands.(E, F) Representative flow cytometry plots (E) and quantitative analysis (F) of PB cells in the spleen.(G, H) Representative flow cytometry plots (G) and quantitative analysis (H) of PB cells in the submandibular glands.Data are presented as mean ± SEM. Statistical significance was determined by [please specify the statistical test used, e.g., one-way ANOVA followed by Tukey's post-hoc test. * p < 0.05, ** p < 0.01, *** p < 0.001


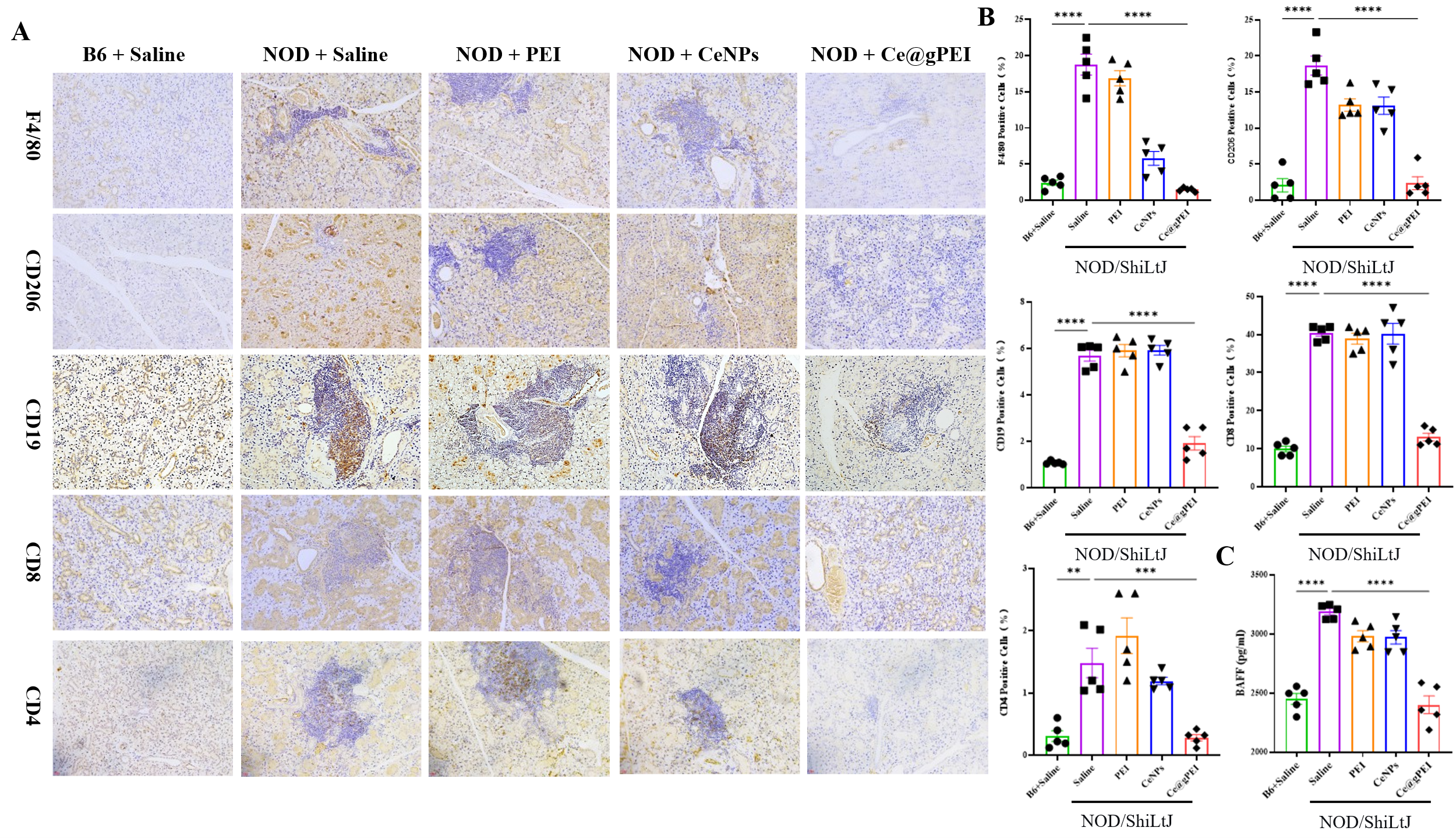


**Figure S10.** Immunohistochemical analysis and serum BAFF level in NOD mice following various treatments. (A) Representative immunohistochemical images of spleen sections from different experimental groups. (B) Quantitative analysis of positive staining area from immunohistochemistry. (C) Serum BAFF concentration measured by ELISA. * *P* < 0.05, ** *P* < 0.01, *** *P* < 0.001.
